# Mannose trimming is the dominant signal for the release of misfolded glycoproteins from ER quality control

**DOI:** 10.1016/j.jbc.2025.110649

**Published:** 2025-08-28

**Authors:** Yun-Ji Shin, Ulrike Vavra, Daniel Maresch, Clemens Grünwald-Gruber, Richard Strasser

**Affiliations:** 1 Institute of Plant Biotechnology and Cell Biology, Department of Biotechnology and Food Sciences, BOKU University, Vienna, Austria; 2 Core Facility Mass Spectrometry, BOKU University, Vienna, Austria

**Keywords:** cell biology, endoplasmic reticulum (ER), endoplasmic reticulum-associated protein degradation (ERAD), glycoprotein, N-linked glycosylation, plant, protein degradation

## Abstract

N-glycosylation is essential for protein folding in the endoplasmic reticulum (ER). Glycan attachment facilitates the binding of newly synthesized polypeptides to calnexin and calreticulin, two ER-resident lectins that act as chaperones and promote folding. The regulatory mechanism underlying this process is dictated by the glycan composition, and this study has elucidated the function of mannose trimming in the release of misfolded glycoprotein from ER quality control and subsequent transfer to ER-associated degradation (ERAD) in plants. Coimmunoprecipitation experiments were performed to investigate the interaction between the ERAD substrate SUBEX-C57Y and ER-resident lectins, providing evidence for a specific interaction with calnexin. Furthermore, the results show that the expression of calnexin and calreticulin can specifically inhibit the degradation of glycosylated ERAD substrates. However, overexpression of the α-mannosidase MNS4 was shown to overcome lectin-dependent accumulation and promote degradation *via* ERAD. MS-based analysis of released N-glycans and jack bean α-mannosidase-digested glycopeptides revealed the presence of monoglucosylated N-glycans even after mannose trimming. This finding is consistent with the observation that calnexin remained associated with SUBEX-C57Y after mannose removal from the C-branch of the N-glycan. In contrast, specific removal of the first α1,2-linked mannose from the A-branch or a block of glucose trimming of Glc3Man9GlcNAc2 prevented the interaction with calnexin. In conclusion, the study provides fundamental insights into the specific role of glycan residues for the interdependence between lectin-based ER quality control and glycan-dependent ERAD in plants.

Endoplasmic reticulum-associated degradation (ERAD) of proteins is a multistep process that eliminates aberrant proteins to prevent their accumulation in the secretory pathway or secretion of potentially harmful variants. In the first clearance step, terminally misfolded proteins that carry, for example, a lesion in a luminal region of the protein are recognized by endoplasmic reticulum (ER)-resident proteins and diverted away from futile protein folding cycles. In the next step, the ERAD substrates are targeted to a membrane embedded E3 ubiquitin ligase complex and subsequently retrotranslocated to the cytoplasm, followed by ubiquitination and proteasomal degradation. Although these steps are well described in mammals and yeast (1), the molecular mechanisms that terminate unproductive protein folding attempts in the ER and lead to the destruction of misfolded proteins in plants are currently not well understood (2).

A huge number of proteins that enter the secretory pathway are glycoproteins and carry a covalently linked glycan attached to an amino acid side chain (3). N-glycosylation is initiated in the ER by the transfer of a preassembled oligosaccharide to an asparagine residue present in the consensus sequence Asn-X-Ser/Thr (X any amino acid except proline) of a nascent polypeptide chain (4). The attached N-glycans play crucial roles in folding of many diverse proteins and regulate ER quality control (ERQC) processes and the degradation of misfolded glycoproteins by ERAD (5). Previous studies reported that glycosylated ERAD substrates like *Arabidopsis thaliana* BRI1-5 or SUBEX-C57Y, a misfolded form of the extracellular domain from the atypical receptor kinase STRUBBELIG (6), display considerable amounts of monoglucosylated N-glycans (7, 8). Glycoproteins carrying such N-glycans are known to associate with the lectin chaperones calnexin (CNX) or calreticulin (CRT) that are essential proteins of the CNX/CRT cycle. The two chaperones hinder the aggregation of nonnative glycoproteins and cooperate with folding assistants to enable efficient folding (9, 10). Deglucosylation catalyzed by α-glucosidase II (GCSII) prevents the glycoproteins from association with CNX/CRT. Properly folded proteins are released from ERQC and allowed to exit to the Golgi apparatus and other compartments of the secretory pathway (11). A protein that has not acquired its native conformation, on the other hand, is either reglucosylated by UDP-glucose:glycoprotein glucosyltransferase (UGGT) or sent for degradation. How the cells differentiate between on-pathway folding and futile off-pathway processes is not fully understood (12). Previously it has been proposed that mannose trimming mediated by slow-acting α-mannosidases functions as a timer to terminate unproductive folding processes (13). The occurrence of monoglucosylated oligosaccharides on ERAD substrates in plants suggests a tight interplay of quality control and degradation processes, and raises the question how the misfolded glycoproteins are released from the CNX/CRT cycle.

In mammalian cells, ER-degradation enhancing α-mannosidase-like (EDEM) proteins interact with CNX and EDEM overexpression causes the faster release of misfolded glycoproteins from CNX and promotes ERAD of nonmembrane bound misfolded glycoproteins (14, 15). In *Schizosaccharomyces pombe*, mannose trimming from the B- and C-branches of the oligomannosidic N-glycan (Fig. 1*A*) reduces GCSII-mediated deglucosylation (16) and thus enables the monoglucosylated N-glycan to interact with CNX/CRT for a prolonged time. The mannose trimming is likely performed by the subsequent action of EDEM1 to EDEM3 in mammals (17, 18). *Arabidopsis* has two EDEM homologs, termed MNS4 and MNS5, which are involved in ERAD and promote the hydrolysis of a terminal mannose residue from the C-branch of the oligomannosidic N-glycan (19, 20). The resulting exposed α1,6-linked mannose (Fig. 1*A*) is recognized as degradation signal by the mannose binding lectin OS9, and the ERAD substrate is targeted to the HRD1 complex for subsequent degradation (21, 22, 23, 24). *Arabidopsis* has two HRD1 E3 ubiquitin ligases (HRD1A and HRD1B) which redundantly function in ERAD (25). MNS4 and MNS5 are the only two class I α-mannosidases required for ERAD of misfolded glycoproteins in plants (19, 26), and it is unclear whether they contribute to the termination of CNX/CRT interaction with nonnative glycoproteins and promote the release from ERQC like it was shown for human EDEM. To address these questions and better understand the degradation process of terminally misfolded glycoproteins in the ER of plants, we characterized the interaction of ERAD substrates with CNX/CRT and the roles of glucose and mannose trimming. An unexpected persistent interaction of the soluble ERAD substrate SUBEX-C57Y with CNXs was identified, which was independent of mannose trimming on the C-branch of the oligomannosidic N-glycan. Furthermore, the data indicate that the glucose on the A-branch and association with CNX/CRT are important for the solubility of aberrant glycoproteins. Our data provide novel insights into this poorly understood process in plants and highlight fundamental differences between humans and plants.

**Figure 1:**
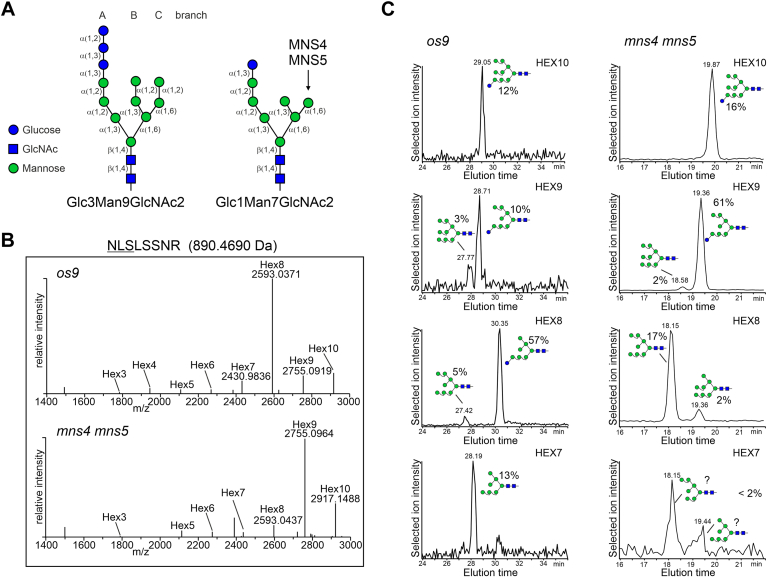
**SUBEX-C57Y from *Arabidopsis* ERAD mutants carries increased amounts of monoglucosylated N-glycans.***A*, illustration of the unprocessed N-linked oligosaccharide (Glc3Man9GlcNAc2) and the MNS4/MNS5-processed N-glycan (Glc1Man7GlcNAc2). The monosaccharides, their linkages, the three branches (*A*, *B*, and *C*) and the mannose residue that is cleaved off by MNS4/MNS5 are indicated. The symbol nomenclature is according to the Consortium for Functional Glycomics. *B*, LC-ESI-MS analysis of the glycopeptide 2 (NLSLSSNR) from SUBEX-C57Y-GFP expressed in *Arabidopsis os9* and *mns4 mns5* mutants. *C*, N-glycans from purified SUBEX-C57Y-GFP were released and analyzed by PGC-LC-ESI-MS. The assignment of structural isomers was based on reference glycans as described in detail previously (78). Due to the use of different gradients, the elution times of distinct N-glycans differs in *os9* and *mns4 mns5*. The HEX7 peaks in *msn4 mns5* could not be unequivocally assigned due to the low signal intensity and broad peaks, and the indicated glycan structures are therefore marked with a question mark. ERAD, ER-associated degradation; LC-ESI-MS, liquid chromatography-electrospray ionization-mass spectrometry; PGC-LC-ESI-MS, porous graphitic carbon chromatography with mass spectrometric

## Results

### The misfolded ERAD substrate SUBEX-C57Y forms a complex with calnexin and MNS4/MNS5

Previously, we showed that the luminal ERAD substrate SUBEX-C57Y fused to GFP (SUBEX-C57Y-GFP) carries glycopeptides with N-glycans corresponding to monoglucosylated oligomannosidic structures (*e.g.* Glc_1_Man_9_) when transiently expressed in WT *Nicotiana benthamiana* plants (7). To further analyze the role of N-glycans, we purified SUBEX-C57Y-GFP from the *Arabidopsis mns4 mns5* double mutant which has a defect in mannose trimming (19) and from the *Arabidopsis os9* mutant which lacks a functional OS9 lectin that binds to the glycan degradation signal generated by MNS4/MNS5 (21). In both mutants, ERAD of glycoproteins is blocked and misfolded SUBEX-C57Y-GFP accumulates (7). The liquid chromatography-electrospray ionization-mass spectrometry (LC-ESI-MS) analysis of SUBEX-C57Y glycopeptides derived from *mns4 mns5* showed that the major peak on N-glycosylation sites 1 and 2 corresponds to a Hex_9_HexNAc_2_ structure (Fig. 1*B*, S1 and Table 1). On site 3, Hex_9_HexNAc_2_ was the second most abundant peak after Hex_10_HexNAc_2_, which was the most abundant peak. In contrast, Hex_8_HexNAc_2_ was most abundant on all three N-glycosylation sites of SUBEX-C57Y-GFP derived from the *os9* mutant. The additional hexose on the *mns4 mns5* derived SUBEX-C57Y-GFP glycopeptides is consistent with the previously determined mannose trimming activity of *Arabidopsis* MNS4/MNS5 (8, 19). An isomer-specific analysis of released N-glycans by porous graphitic carbon chromatography with mass spectrometric (PGC-LC-ESI-MS) revealed further that the predominant peaks of SUBEX-C57Y-GFP purified from *os9* and *mns4 mns5* eluted different from defined Man_9_GlcNAc_2_ and Man_8_GlcNAc_2_ N-glycan standards (Fig. 1*C*). The isobaric Hex_9_HexNAc_2_ and Hex_8_HexNAc_2_ structures from *os9* and *mns4 mns5* thus contain one or more hexose residue that are distinct from the mannoses present on the B- and C-branches of Man_9_GlcNAc_2_ or Man_8_GlcNAc_2_ (Fig. 1*A*). Consequently, they were assigned to monoglucosylated structures, such as Glc_1_Man_8_GlcNAc_2_ (Hex_9_HexNAc_2_ peak in *mns4 mns5*) and Glc_1_Man_7_GlcNAc_2_ (Hex_8_HexNAc_2_ peak in *os9*) (Fig. 1*C*). This shows that monoglucosylated N-glycans are the most abundant glycoforms (approximately 60%) on the SUBEX-C57Y ERAD substrate.

**Table 1:** Relative amounts of glycans from LC-ESI-MS-analysis of SUBEX-C57Y glycopeptides

Glycan composition	Site 1 (NIT)		Site 2 (NLS)		Site 3 (NTS)	
	*os9*	*mns4 mns5*	*os9*	*mns4 mns5*	*os9*	*mns4 mns5*
HexNAc1	3.1	3.6	1.6	2.5	0.9	0
Hex2HexNAc2 (HEX2)	0.3	0.1	0	0	0.1	0
Hex3HexNAc2 (HEX3)	0.6	0.3	1.0	0.7	0.6	0
Hex4HexNAc2 (HEX4)	1.3	0.6	3.0	0	1.3	0.8
Hex5HexNAc2 (HEX5)	1.6	1.2	2.5	4.1	2.3	1.7
Hex6HexNAc2 (HEX6)	2.6	1.4	2.5	3.4	3.2	3.1
Hex7HexNAc2 (HEX7)	16.4	2.8	6.9	3.2	8.4	3.2
Hex8HexNAc2 (HEX8)	63.7	12.7	61.7	7.4	35.0	8.8
Hex9HexNAc2 (HEX9)	5.0	56.0	9.7	58.6	19.3	36.2
Hex10HexNAc2 (HEX10)	5.5	21.3	11.2	20.0	28.8	46.2
total (%)	100	100	100	100	100	100

**Figure 2:**
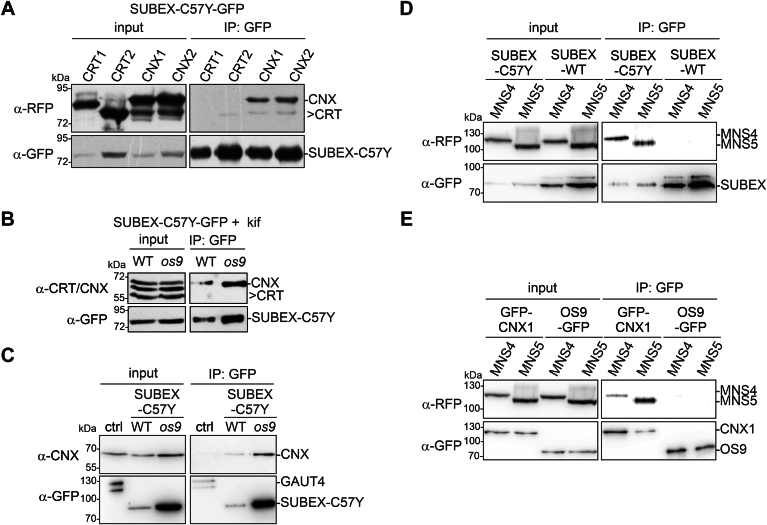
**CNXs interact with the luminal ERAD substrate SUBEX-C57Y.***A*, SUBEX-C57Y-GFP was transiently expressed in *Nicotiana benthamiana* leaves with RFP-tagged CNX or CRT variants, purified by immunoprecipitation and copurified RFP-tagged proteins were detected by immunoblotting. *B*, SUBEX-C57Y-GFP was purified from *Arabidopsis* wild-type (WT) or the *os9* mutant and endogenous CNX/CRT were detected with a specific antibody that binds to both lectins. To increase the amount of SUBEX-C57Y-GFP in WT, WT and *os9* seedlings were incubated with 100 μM kifunensine (kif) for 18 h prior to protein extraction. *C*, SUBEX-C57Y-GFP was purified from *Arabidopsis* WT or *os9* plants. GAUT4-GFP (control - ctrl) was purified from *Arabidopsis* WT plants, and immunoblots were probed using anti-CNX antibodies that do not bind to CRT. *D*, SUBEX-C57Y-GFP or SUBEX-WT-GFP was transiently coexpressed with RFP-tagged MNS4 or MNS5, purified by immunoprecipitation and tagged proteins were detected by immunoblotting. *E*, GFP-CNX1 or OS9-GFP was transiently expressed together with RFP-tagged MNS4 or MNS5 variants. GFP-tagged proteins were immunoprecipitated and copurified proteins detected by immunoblotting. CNX, calnexin; CRT, calreticulin; ERAD, ER-associated degradation; GAUT4, galacturonosyltransferase 4; RFP, red fluorescent protein.

The presence of monoglucosylated oligomannosidic N-glycans prompted us to investigate whether there is an interaction between the lectins CNX/CRT and glycosylated ERAD substrates like SUBEX-C57Y. To this end, we transiently expressed SUBEX-C57Y-GFP together with red fluorescent protein (RFP)-tagged CNX or CRT variants (*Arabidopsis* CRT1, CRT2, CNX1, and CNX2) in leaves of *N. benthamiana*. The GFP-tagged ERAD substrate was purified by binding to GFP-Trap beads and the copurification of RFP-tagged CNX or CRT was monitored by immunoblotting (Fig. 2*A*). Although SUBEX-C57Y-GFP poorly interacted with *Arabidopsis* CRT1-RFP and CRT2-RFP, RFP-CNX1 and RFP-CNX2 were copurified with SUBEX-C57Y-GFP. Similarly, when SUBEX-C57Y-GFP was purified from *Arabidopsis* WT or the *os9* mutant, the endogenous CNX proteins were associated with the SUBEX-C57Y-GFP ERAD substrate but not CRT1 and CRT2 (Fig. 2*B*). The amount of copurified CNX was dependent on the amount of SUBEX-C57Y and specific as no interaction was detected with the control protein, galacturonosyltransferase 4 fused to GFP (GAUT4-GFP) (Fig. 2*C*) which harbors five N-glycosylation sites. Overall, this finding is consistent with the presence of monoglucosylated N-glycans, and an earlier study that showed the specific binding of CNX to the ERAD substrate BRI1-5, which is a heavily glycosylated misfolded form of the membrane anchored brassinosteroid receptor BRI1 (27).

Next, we tested whether SUBEX-C57Y-GFP interacts with MNS4 and MNS5. As a control for a specific interaction, we expressed MNS4 and MNS5 with SUBEX-WT-GFP (Fig. S2), which carries the properly folded extracellular domain and is not subjected to ERAD (7). MNS4 and MNS5 were bound to SUBEX-C57Y-GFP, whereas SUBEX-WT-GFP did not show any binding to MNS4 and MNS5 (Fig. 2*D*). Having shown the binding of SUBEX-C57Y-GFP to CNX1/CNX2 and to MNS4/MNS5, we examined whether there is a complex formation between MNS4/MNS5 and CNX1. GFP-CNX1 was associated with MNS4-RFP and MNS5-RFP (Fig. 2*E*). By contrast, there was virtually no binding of MNS4/MNS5 to the lectin OS9 (Fig. 2*E*), which has previously been shown to interact with the ERAD substrate SUBEX-C57Y-GFP (7).Together these data show the formation of a specific complex between the ERAD substrate SUBEX-C57Y-GFP, CNX1/CNX2, and MNS4/MNS5.

### CNX/CRT overexpression leads to the accumulation of glycosylated ERAD substrates

**Figure 3:**
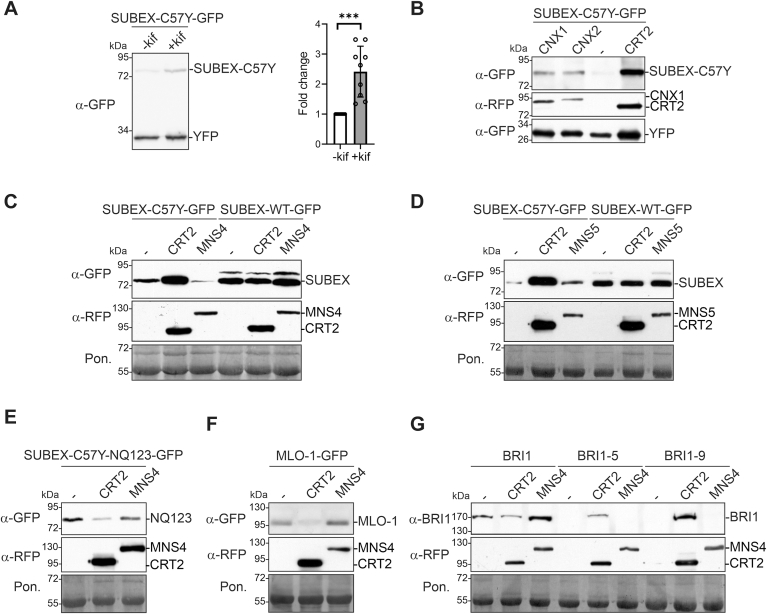
**CNX or CRT coexpression prevents ERAD of misfolded glycoproteins.***A*, SUBEX-C57Y-GFP was transiently expressed in *Nicotiana benthamiana* leaves with kifunensine (+kif, 20 μM) and detected by immunoblotting. For quantification of the increase in SUBEX-C57Y levels, the SUBEX-C57Y-GFP signal was normalized to the YFP signal detected on the same blot with the same antibody. Error bar represents mean ± SD (n = 9, “∗∗∗”, *p* < 0.001 according to an unpaired Student’s *t* test). *B*, SUBEX-C57Y-GFP was transiently expressed in *N. benthamiana* leaves with RFP-tagged CNX1, CNX2, or CRT2. *C*-*G*, SUBEX-C57Y-GFP, SUBEX-WT-GFP, SUBEX-C57Y-NQ123-GFP, MLO-1-GFP, or BRI1-variants were transiently expressed in *N. benthamiana* leaves with RFP-tagged CRT2 or MNS4 and subjected to immunoblotting. Ponceau S (Pon.) staining is used as a loading control. CNX, calnexin; CRT, calreticulin; ERAD, ER-associated degradation; RFP, red fluorescent protein.

During the copurification experiments, it was observed that CNX or CRT coexpression increased the steady-state amounts of SUBEX-C57Y-GFP suggesting that protein degradation is delayed in the presence of a monoglucosylated N-glycan binding lectin. To determine the impact of CNX/CRT on the ERAD substrate more precisely, we used a SUBEX-C57Y-GFP expression vector comprising an additional expression cassette for a cytosolic YFP. YFP expression serves as an internal control to account for potential variations resulting from the infiltration process or protein extraction (Fig. S2). GFP fused to the ERAD substrate and cytosolic YFP can be detected on the same immunoblot membrane using a GFP-specific antibody. To test this approach, we expressed SUBEX-C57Y-GFP in *N. benthamiana* leaves, both in the presence and absence of the α-mannosidase inhibitor kifunensine that blocks the glycan-dependent ERAD of misfolded glycoproteins (7). As expected, SUBEX-C57Y-GFP was significantly stabilized by kifunensine (Fig. 3*A*). Likewise, the coexpression of CNX1, CNX2, CRT1, or CRT2 resulted in elevated SUBEX-C57Y-GFP levels compared to the control (Fig. 3*B* and S3). The stabilizing effect was contingent upon the level of CNX/CRT expression, with CRT2-RFP exhibiting a more pronounced effect in comparison to the CNX variants. In addition to an increased amount on immunoblots, the stabilizing effect of CRT2 and CNX1 overexpression was clearly detectable by an increased fluorescence signal of SUBEX-C57Y-GFP in the ER of leaf epidermal cells (Fig. S4). The impact of CRT2 was specific for the misfolded glycoprotein, as no changes were observed for the glycosylated native SUBEX-WT-GFP form (Fig. 3, *C* and *D*) as well as for the nonglycosylated misfolded SUBEX-C57Y-NQ123-GFP variant (Fig. 3*E*), where all N-glycosylation sites are mutated (7). In accordance with this finding, the amount of a mutant form of barley powdery mildew resistance O (MLO-1-GFP) protein, which is a nonglycosylated ERAD substrate (28, 29, 30), was not increased by CRT2 expression (Fig. 3*F*). On the other hand, the misfolded glycosylated ERAD substrates BRI1-5 (27) and BRI1-9 (31) were stabilized by CRT2 coexpression (Fig. 3*G*). The findings demonstrate that the overexpression of CNX/CRT variants leads to the accumulation of misfolded glycoproteins.

### MNS4 overexpression prevents the CNX/CRT-mediated SUBEX-C57Y accumulation

**Figure 4:**
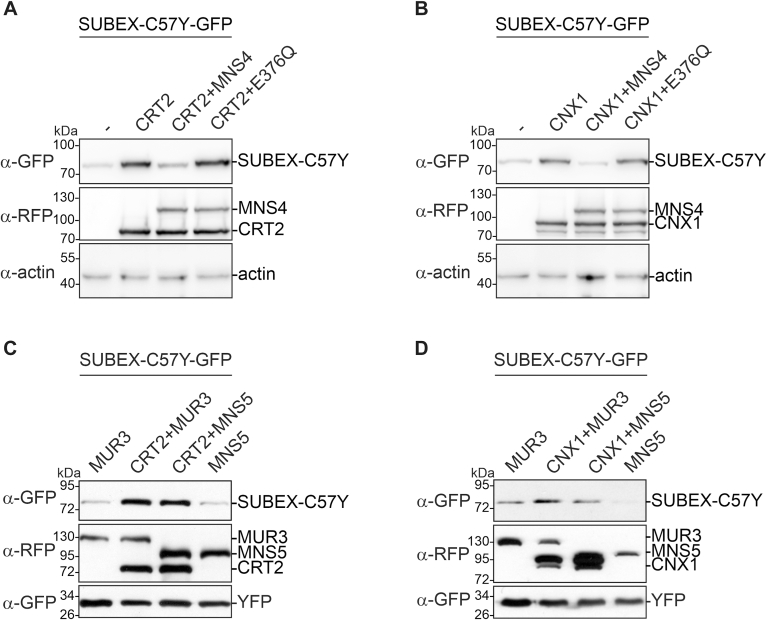
**MNS4 overexpression promotes ERAD of SUBEX-C57Y.***A*, SUBEX-C57Y-GFP was transiently expressed in *Nicotiana benthamiana* leaves without any additional protein (−), with CRT2-RFP (CRT2), with a combination of CRT2-RFP and MNS4-RFP (CRT2+MNS4) or CRT2-RFP and MNS4-E376Q-RFP (active site mutant of MNS4). The detection of actin was used as a loading control. *B*, SUBEX-C57Y-GFP was transiently expressed in *N. benthamiana* leaves without any additional protein (−), with RFP-CNX1 (CNX1), with a combination of RFP-CNX1 and MNS4-RFP (CNX1+MNS4) or RFP-CNX1 and MNS4-E376Q-RFP (CNX1+E376Q). *C*, SUBEX-C57Y-GFP was transiently expressed in *N benthamiana* leaves with the unrelated control protein MUR3-RFP (MUR3), with CRT2-RFP and MUR3-RFP (CRT2+MUR3), with a combination of CRT2-RFP and MNS5-RFP (CRT2+MNS5) or with MNS5-RFP alone (MNS5). *D*, SUBEX-C57Y-GFP was transiently expressed with the unrelated control protein MUR3-RFP (MUR3), RFP-CNX1 and MUR3-RFP (CNX1+MUR3), RFP-CNX1 and MNS5-RFP (CNX1+MNS5) or MNS5-RFP (MNS5). The detection of YFP was used as a control for both expression and protein extraction. CNX, calnexin; CRT, calreticulin; ERAD, ER-associated degradation; RFP, red fluorescent protein; YFP, yellow fluorescent protein.

Mammalian EDEM1 plays a role in the release of glycoprotein ERAD substrates from CNX (14, 15). Overexpression of either MNS4 or MNS5 enhanced the severe growth defect of *bri1-5* plants (19) indicating that their α-mannosidase activity accelerates misfolded glycoprotein degradation. MNS4-RFP coexpression reduced the amount of SUBEX-C57Y-GFP (Fig. 3*C*) and abrogated the stabilizing effect of both CRT2 and CNX1 on SUBEX-C57Y-GFP (Fig. 4, *A* and *B*). Notably, coexpression of the MNS4 active-site mutant MNS4-E376Q did not reduce SUBEX-C57Y-GFP levels indicating that mannose trimming is necessary to promote SUBEX-C57Y degradation. Unlike MNS4-RFP, MNS5-RFP had little impact on the degradation of this misfolded protein (Figs. 3*D* and 4, *C* and *D*). Furthermore, MNS4 diminished the stabilizing effect of CRT2 on BRI1-5 (Fig. S5), thereby confirming that MNS4 activity facilitates the degradation of glycan-dependent ERAD substrates and overcomes the CNX/CRT-dependent accumulation.

To block the degradation at a later step of the pathway, we expressed a catalytically inactive variant of the cytosolic *Arabidopsis* AAA ATPase CDC48A (CDC48-QQ). CDC48 is indispensable for the retrotranslocation process, facilitating the extraction of misfolded ubiquitinated proteins from the ER membrane (32). The mutant CDC48-QQ variant carries an amino acid exchange in each of the two ATPase domains (E308Q and E581Q), and its overexpression was previously shown to stabilize misfolded MLO-1 and SUBEX-C57Y (24, 28). The coexpression of CDC48-QQ resulted in an increase in SUBEX-C57Y-GFP levels and other misfolded proteins, as observed on immunoblots (Fig. S6). In the presence of RFP-CDC48-QQ and MNS4-RFP, SUBEX-C57Y-GFP accumulated (Fig. S6), which is consistent with the function of CDC48 after the generation of the glycan degradation signal and downstream of the HRD1 core complex (33).

**Figure 5:**
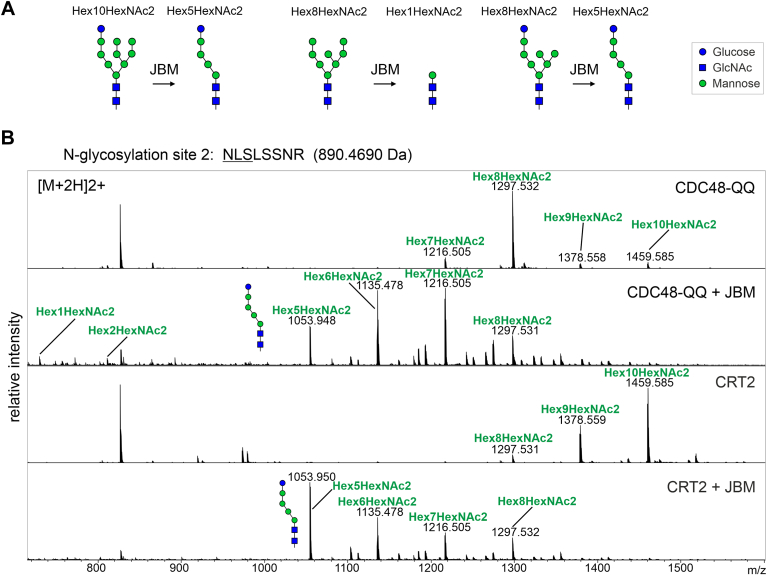
**SUBEX-C57Y carries monoglucosylated N-glycans when the dislocation is blocked.***A*, illustration of jack bean α-mannosidase (JBM) activity on different N-glycan substrates. *B*, LC-ESI-MS analysis of the SUBEX-C57Y glycopeptide carrying N-glycosylation site 2. SUBEX-C57Y-GFP was transiently coexpressed in *Nicotiana benthamiana* leaves with RFP-CDC48-QQ (CDC48-QQ) or CRT2-RFP (CRT2), purified, followed by proteolytic digestion, digested with JBM (+JBM) and subjected to LC-ESI-MS analysis. The most prominent N-glycan peaks are labeled and the Hex5HexNAc2 structure is illustrated. CRT, calreticulin; LC-ESI-MS, liquid chromatography-electrospray ionization-mass spectrometry; RFP, red fluorescent protein.

To confirm this finding and characterize potential differences in N-glycan structures at different stages of the pathway, we expressed SUBEX-C57Y-GFP together with CRT2-RFP or RFP-CDC48-QQ, purified the misfolded protein and subjected tryptic peptides from SUBEX-C57Y-GFP to LC-ESI-MS analysis. CRT2 coexpression resulted in the presence of Hex_10_HexNAc_2_ as the most abundant N-glycan on all three SUBEX-C57Y-GFP N-glycosylation sites (Fig. S7). Hex_10_HexNAc_2_ structures are indicative of the presence of monoglucosylated N-glycans corresponding to Glc_1_Man_9_GlcNAc_2_. SUBEX-C57Y-GFP stabilized by RFP-CDC48-QQ expression, on the other hand, displayed Hex_8_HexNAc_2_ as a major N-glycan. This structure corresponds either to Man_8_GlcNAc_2_ or Glc_1_Man_7_GlcNAc_2_. To differentiate between those two structures, we digested SUBEX-C57Y with jack bean α-mannosidase (JBM) which trims α-linked mannoses from the oligomannosidic N-glycan unless the ones from the A-branch when they are capped with at least one glucose residue (Fig. 5*A*). Absence of glucose on the A-branch results in trimming to Hex_1_HexNAc_2_ (33, 34). When coexpressed with RFP-CDC48-QQ, LC-ESI-MS analysis of SUBEX-C57Y glycopeptide 2 digested with JBM showed the presence of Hex_5-8_HexNAc_2_ peaks and only low amounts of Hex_1_HexNAc_2_ (Fig. 5*B*). Consequently, this finding indicates that substantial amounts of monoglucosylated Glc_1_Man_7_GlcNAc_2_ with a terminal α1,6-linked mannose residue on the ERAD substrate are present at a late stage when the CDC48-mediated protein dislocation to the cytosol was impaired.

### Mannose trimming does not release SUBEX-C57Y from CNX

**Figure 6:**
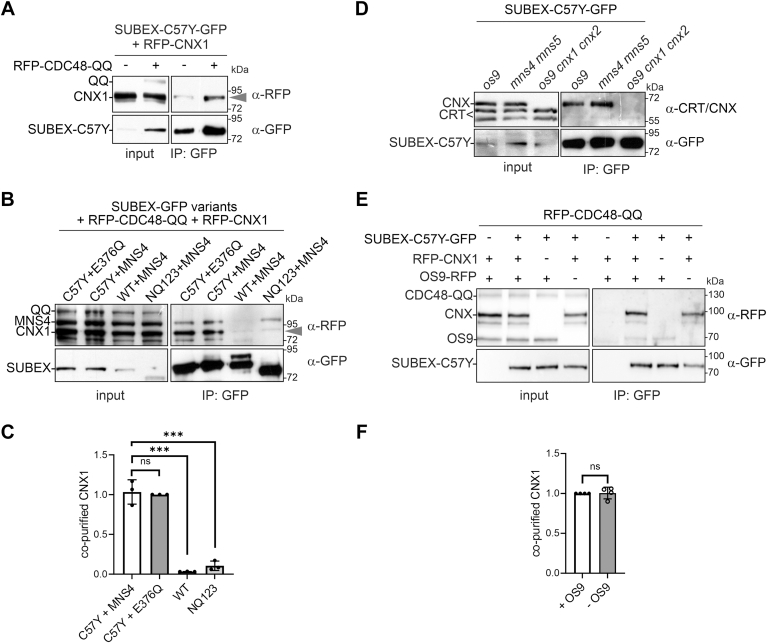
**MNS4 activity does not impede the association between CNX1 and SUBEX-C57Y.***A*, SUBEX-C57Y-GFP was transiently expressed in *Nicotiana benthamiana* leaves with RFP-tagged CNX1 (RFP-CNX1) in the absence (−) or presence (+) of RFP-CDC48-QQ, purified by immunoprecipitation and analyzed by immunoblotting. The *arrowhead* marks the position of RFP-CNX1. *B*, SUBEX variants (C57Y: SUBEX-C57Y-GFP; WT: SUBEX-WT-GFP; NQ123: SUBEX-C57Y-NQ123-GFP) were transiently coexpressed with RFP-CDC48-QQ, RFP-CNX1 and active MNS4-RFP (MNS4) or inactive MNS4-E376Q-RFP (E376Q), purified by binding to GFP-Trap beads and subjected to immunoblotting. The *arrowhead* marks the position of RFP-CNX1. *C*, quantification of the copurified RFP-CNX1. The RFP-CNX1 signal on immunoblots was normalized to purified SUBEX-C57Y-GFP. Data represent means ± SD (n = 3, “ns” indicates that the difference is not significant, *p* > 0.05; “∗∗∗”, *p* < 0.001 according to an unpaired Student’s *t* test). *D*, SUBEX-C57Y-GFP was purified from *Arabidopsis* mutants and subjected to immunoblotting to detect copurified CNX or CRT. The used anti-CRT/CNX antibody detects CRT (faster migrating) and CNX variants. *E*, SUBEX-C57Y-GFP was coexpressed with RFP-CDC48-QQ, RFP-CNX1, and OS9-RFP in *N. benthamiana* leaves, purified by binding to GFP-Trap beads and subjected to immunoblotting to detect copurified proteins. *F*, quantification of the copurified RFP-CNX1. The RFP-CNX1 signal on immunoblots was normalized to purified SUBEX-C57Y-GFP. Error bar represents mean ± SD (n = 4, “ns” indicates that the difference is not significant according to an unpaired Student’s *t* test). CNX, calnexin; CRT, calreticulin; RFP, red fluorescent protein.

Monoglucosylated N-glycans would allow a prolonged interaction with CNX/CRT even after mannose trimming on the B- and C-branches. Such an interaction implies that mannose trimming by MNS4 does not play a role in the release of the protein from the CNX/CRT cycle which is contrary to findings for the function of mammalian EDEM (14, 15). To investigate the association with the lectin CNX during different ERAD steps, we analyzed the amount of RFP-CNX1 that is copurified with SUBEX-C57Y-GFP when ERAD is blocked at a late step by RFP-CDC48-QQ expression. In the presence of RFP-CNX1 or RFP-CDC48-QQ, SUBEX-C57Y-GFP protein levels were increased and the amount of copurified RFP-CNX1 correlated with the amount of purified SUBEX-C57Y-GFP (Fig. 6*A*). Upon coexpression of MNS4-RFP or the catalytically inactive MNS4-E376Q-RFP variant, essentially the same amounts of RFP-CNX1 were copurified (Fig. 6, *B* and *C*). Together with the glycopeptide analysis that showed Glc_1_Man_7_GlcNAc_2_ glycans in the presence of CDC48-QQ (Fig. 5*B*), these data show that RFP-CNX1 and SUBEX-C57Y-GFP are still associated after mannose trimming. The correctly folded SUBEX-WT-GFP protein, which was included as a control in the copurification experiment, neither interacted with RFP-CNX1 nor with MNS4-RFP. This was confirmed by mass spectrometry (MS)-based analysis of proteins from *N. benthamiana* that copurified with SUBEX-C57Y-GFP in comparison to the control SUBEX-WT. Endogenous CNX was associated with SUBEX-C57Y-GFP, whereas the levels of copurified CRT were much lower and comparable to the control (Table S3). The nonglycosylated misfolded SUBEX-C57Y-NQ123-GFP displayed interaction with MNS4-RFP (Fig. 6*B*) indicating that MNS4 may bind to certain regions of misfolded proteins independently of N-glycans. By contrast, RFP-CNX1 interaction with SUBEX-C57Y-NQ123-GFP was hardly detected (Fig. 6, *B* and *C*).

Next, purification of SUBEX-C57Y-GFP from *Arabidopsis os9*, which displays the free α1,6-mannose on the C-branch, and the *mns4 mns5* double mutant, where this α1,6-mannose is inaccessible due to the presence of an additional α1,2-mannose (Fig. 1, *B* and *C*), was conducted. Native CNX was associated with the ERAD substrate in comparable amounts (Fig. 6*D*). This indicates that OS9 has little impact on CNX association. To examine whether OS9 overexpression would titrate the misfolded ERAD substrate away from CNX, we coexpressed SUBEX-C57Y with CNX1 in the absence or presence of OS9. To block the degradation of the ERAD substrate, CDC48-QQ was also coexpressed. In the presence of OS9, the levels of CNX1 associated with SUBEX-C57Y were essentially equivalent to those observed in its absence, indicating that there is no competition of OS9 and CNX1 for binding to the ERAD substrate (Fig. 6, *E* and *F*). The combined data show that the interaction with CNX1 is dependent on the presence of monoglucosylated N-glycans on the misfolded proteins, and that neither MNS4-mediated mannose trimming nor OS9 expression diminish this interaction with CNXs.

### Monoglucosylated N-glycans are critical for CNX interaction and subsequent ERAD

**Figure 7:**
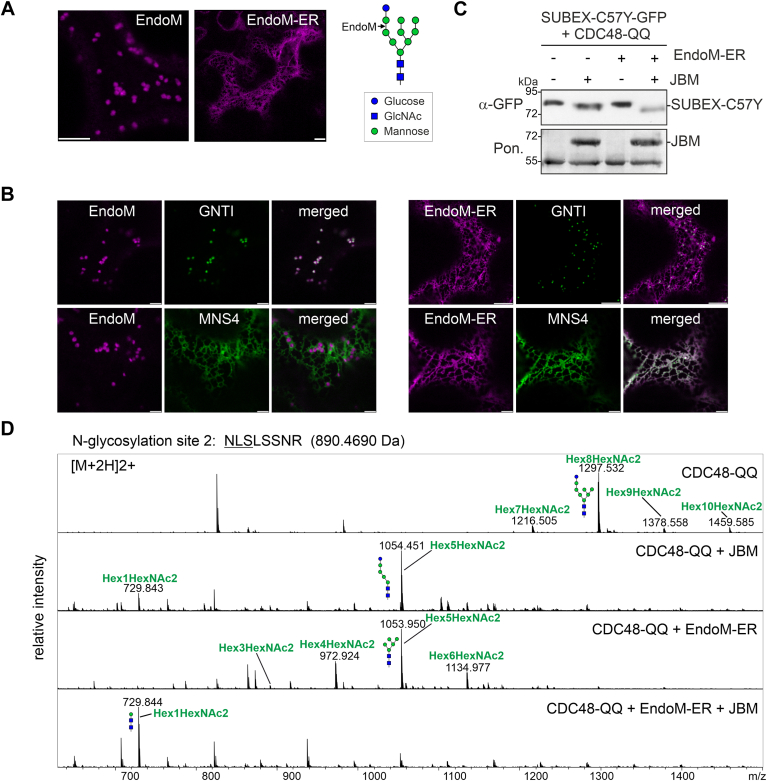
**Endomannosidase M (EndoM) digestion confirms the presence of glucose on the A-branch.***A*, the N-glycan processing activity of EndoM is indicated in the illustration. Subcellular localization of EndoM and EndoM-ER carrying the C-terminal KDEL peptide for Golgi to ER-retrieval. The scale bars represent 10 μm. *B*, EndoM-RFP and EndoM-ER-RFP, respectively, were coexpressed with the Golgi marker GNTI-GFP or the ER-resident MNS4-GFP. The scale bars represents 10 μm. *C*, SUBEX-C57Y-GFP was transiently expressed with RFP-CDC48-QQ and without (-EndoM-ER) or with EndoM-ER (+EndoM-ER) followed by JBM digestion prior to immunoblotting. *D*, LC-ESI-MS analysis of the SUBEX-C57Y glycopeptide carrying N-glycosylation site 2. SUBEX-C57Y-GFP was transiently expressed with RFP-CDC48-QQ and with/without EndoM-ER-RFP, purified, followed by proteolytic digestion and the glycopeptide was digested with JBM (+JBM) prior to LC-ESI-MS. The most prominent N-glycan peaks are labeled and illustrations are shown for Hex_8_HexNAc_2_, Hex_5_HexNAc_2_ and Hex_1_HexNAc_2_. ER, endoplasmic reticulum; JBM, jack bean α-mannosidase; LC-ESI-MS, liquid chromatography-electrospray ionization-mass spectrometry; RFP, red fluorescent protein

To further examine the role of CNX/CRT and the monoglucosylated N-glycan for degradation of SUBEX-C57Y-GFP, we transiently expressed a mammalian endomannosidase (EndoM) in *N. benthamiana*. Plants lack EndoM-like enzymes which can remove the Glcα1,3-Man disaccharide from the A-branch of a monoglucosylated N-glycan (35) (Fig. 7*A*). In mammals, EndoM-mediated cleavage of the disaccharide from the A branch provides an alternative processing route to the conventional GCSII-mediated trimming of single glucose residues and prevents the reglucosylation and exposure of monoglucosylated N-glycans to CNX/CRT (36). EndoM is typically found in the Golgi apparatus in mammalian cells (37). To enable ER-localization of EndoM in plants, we attached the HDEL ER-retrieval signal to the C-terminal end of mouse EndoM. Confocal microscopy of RFP-tagged EndoM-ER confirmed the ER-localization, while the EndoM variant lacking the HDEL tetrapeptide was exclusively found in the Golgi (Fig. 7, *A* and *B*). The EndoM-ER expression caused a slight increase in the migration rate of SUBEX-C57Y-GFP upon SDS-PAGE separation and immunoblotting (Fig. 7*C*) which is indicative of N-glycan trimming. Following JBM digestion, an even faster-migrating variant of SUBEX-C57Y-GFP was observed in the presence of EndoM-ER. This indicates that the N-glycans are more extensively processed due to the absence of a glucose residue on the A-branch. MS analysis of JBM-digested glycopeptides from SUBEX-C57Y-GFP coexpressed with EndoM-ER and CDC48-QQ corroborated this finding and revealed a decrease in Hex_5_HexNAc_2_ and an increase in processed Hex_1_HexNAc_2_ structures (Fig. 7*D*).

**Figure 8:**
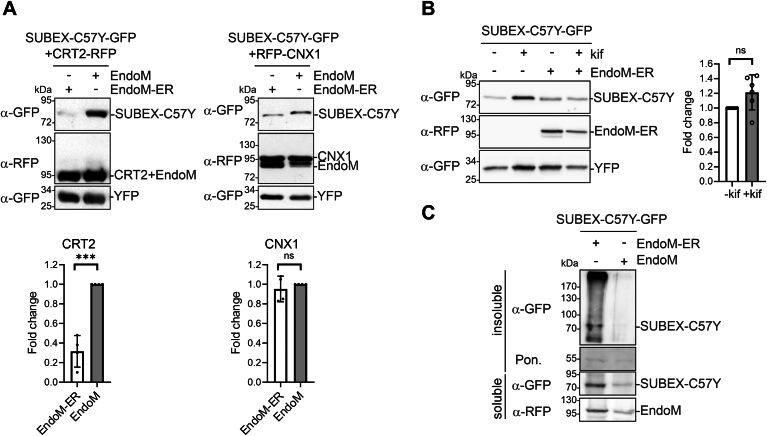
**Removal of the glucose residue from the A branch reduces the amount of SUBEX-C57Y subjected to ERAD.***A*, SUBEX-C57Y-GFP and CRT2-RFP or RFP-CNX1 were transiently expressed in *Nicotiana benthamiana* leaves with EndoM-ER-RFP (Endo-ER) or EndoM-RFP (EndoM) and subjected to immunoblotting. Quantification of the increase in SUBEX-C57Y-GFP signal which was normalized to the YFP expression. Data represent means ± SD (n = 3, “ns” indicates that the difference is not significant, *p* > 0.05; “∗∗∗”, *p* < 0.001 according to an unpaired Student’s *t* test). *B*, SUBEX-C57Y-GFP was transiently expressed with EndoM-ER-RFP in the presence/absence of kifunensine (kif). Quantification was performed by normalizing the SUBEX-C57Y-GFP signal in the presence of EndoM-ER, with or without 100 μM kif incubation, to YFP expression. Data represent means ± SD (n = 3, “ns” indicates that the difference is not significant, *p* > 0.05; “∗∗∗”, *p* < 0.001 according to an unpaired Student’s *t* test). *C*, SUBEX-C57Y-GFP was transiently expressed without (−), with EndoM-ER-RFP (EndoM-ER) or with EndoM-RFP (EndoM), the Triton X-100 soluble and the Triton X-100 insoluble proteins (pellet resuspended in SDS-PAGE sample buffer) were analyzed by immunoblotting. Ponceau S (Pon.) staining is used as a loading control. CNX, calnexin; CRT, calreticulin; EndoM, endomannosidase; ER, endoplasmic reticulum; ERAD, ER-associated degradation; RFP, red fluorescent protein.

In contrast to the Golgi-targeted EndoM, which was used as a control, EndoM-ER abrogated the stabilizing effect of CRT2 on SUBEX-C57Y-GFP (Fig. 8*A*). It is noteworthy that EndoM-ER had a minimal impact when CNX1 was coexpressed with SUBEX-C57Y-GFP (Fig. 8*A*). This shows that the processing of monoglucosylated N-glycans by EndoM-ER specifically interferes with the function of CRT. Furthermore, in the presence of EndoM-ER, no kifunensine-mediated block of glycan-dependent ERAD was observed (Fig. 8*B*). As demonstrated (Fig. 3*A*), a block of ERAD with kifunensine resulted in a 2.7-fold increase in SUBEX-C57Y-GFP amounts. Conversely, coexpression of EndoM-ER in the presence of kifunensine resulted in a negligible increase in the SUBEX-C57Y-GFP signal on immunoblots (Fig. 8*B*). To ascertain whether the solubility of SUBEX-C57Y-GFP is altered when EndoM-ER is expressed, a comparison was conducted between the accumulation in Triton X-100 soluble and insoluble fractions. An enrichment of the SUBEX-C57Y-GFP ERAD substrate was observed in the insoluble fraction when EndoM-ER was coexpressed, indicating an increased propensity for aggregation (Fig. 8*C*).

**Figure 9:**
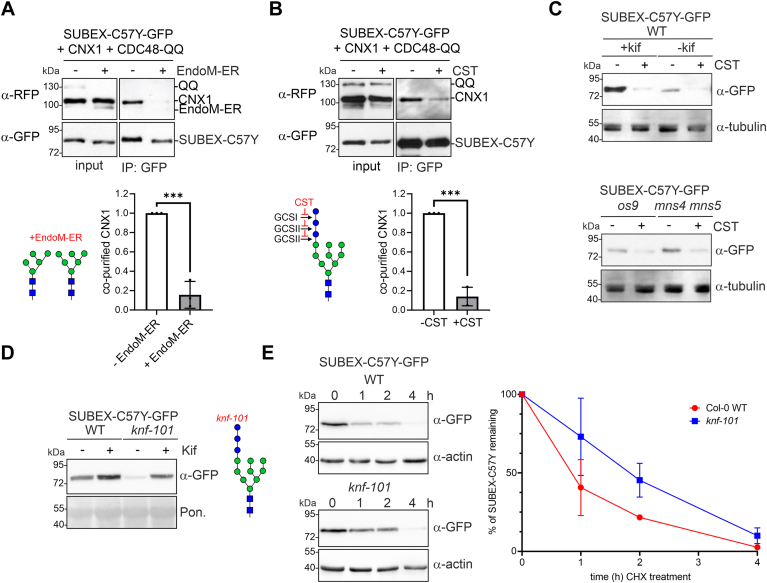
**The interaction of SUBEX-C57Y and CNX1 is dependent on the presence of monoglucosylated N-glycans.***A*, SUBEX-C57Y-GFP, RFP-CDC48-QQ (CDC48-QQ), and RFP-CNX1 (CNX1) were transiently expressed in *Nicotiana benthamiana* leaves with (+) or without (−) EndoM-ER-RFP (EndoM-ER), purified by GFP-Trap purification and subjected to immunoblotting. Quantification of the copurified RFP-CNX1 levels was done by normalization to purified SUBEX-C57Y-GFP. Error bar represents mean ± SD (n = 3, “∗∗∗”, *p* < 0.001 according to an unpaired Student’s *t* test). *B*, SUBEX-C57Y-GFP, RFP-CDC48-QQ, and RFP-CNX1 were transiently expressed with (+) or without (−) 20 μM castanospermine (CST), purified by GFP-Trap purification and subjected to immunoblotting. Quantification of the copurified RFP-CNX1 levels was done by normalization to purified SUBEX-C57Y-GFP. Error bar represents mean ± SD (n = 3, “∗∗∗”, *p* < 0.001 according to an unpaired Student’s *t* test). *C*, *Arabidopsis* seedlings (WT, *os9*, and *mns4 mns5*) were incubated for 24 h with the inhibitors CST (100 μM) and kifunensine (kif, 50 μM) and protein extracts were subjected to immunoblotting. *D*, *Arabidopsis* seedlings (Col-0 and *knf-101*) were incubated for 24 h with kif (50 μM), and protein extracts were subjected to immunoblotting. *E*, degradation of SUBEX-C57Y-GFP in WT and *knf-101* in the presence of 100 μg/ml cycloheximide (CHX). For quantification, the signal from SUBEX-C57Y-GFP was normalized to the actin protein level. Data represent means ± SD (n = 3). CNX, calnexin; EndoM, endomannosidase; ER, endoplasmic reticulum; RFP, red fluorescent protein.

In consideration of the findings, it was hypothesized that the absence of glucose on the A-branch of the N-glycan would result in a reduced interaction with CNX. The presence of EndoM-ER resulted in a significant reduction in the amount of copurified CNX1 (Fig. 9*A*). Monoglucosylated N-glycans are generated immediately following the transfer of the oligosaccharide to the polypeptide by the subsequent action of GCSI and GCSII. To examine the effect of blocked glucose trimming on ERAD of SUBEX-C57Y-GFP, we incubated *Arabidopsis* seedlings with the GCSI/GCSII inhibitor castanospermine (CST). This resulted in a marked diminution of the interaction between SUBEX-C57Y-GFP and RFP-CNX1, in line with the impact of EndoM-ER expression (Fig. 9*B*). Furthermore, SUBEX-C57Y-GFP levels in *Arabidopsis* seedlings incubated with CST for 24 h were consistently lower (Fig. 9*C*). The reduction in SUBEX-C57Y-GFP levels in the presence of CST was also evident in the presence of kifunensine, as well as in *Arabidopsis os9* and *mns4 mns5* mutants. In accordance with these data, confocal microscopy revealed that the amount of fluorescently tagged SUBEX-C57Y was reduced in the ER in the presence of CST, and the retrotranslocation of the ERAD substrate to the cytosol was not observed (Fig. S8).

To gain further insight into the function of the glucose residues on the A-branch, we expressed SUBEX-C57Y-GFP in the *Arabidopsis knf-101* mutant, which carries a hypomorphic allele of GCSI (38) and exhibits elevated levels of glucosylated N-glycans (39). Although SUBEX-C57Y is still subjected to degradation in a glycan-dependent manner in *knf-101* (Fig. 9*D*), the degradation is markedly slower in *knf-101* compared to WT (Fig. 9*E*). In conclusion, the data collectively indicate that an initial impediment to glucose trimming (CST, *knf-101*) or the permanent removal of the last glucose (EndoM-ER) hinders the association with CNX, diverting the protein away from ER quality control processes. This results in altered solubility instead of targeting to ERAD.

## Discussion

**Figure 10:**
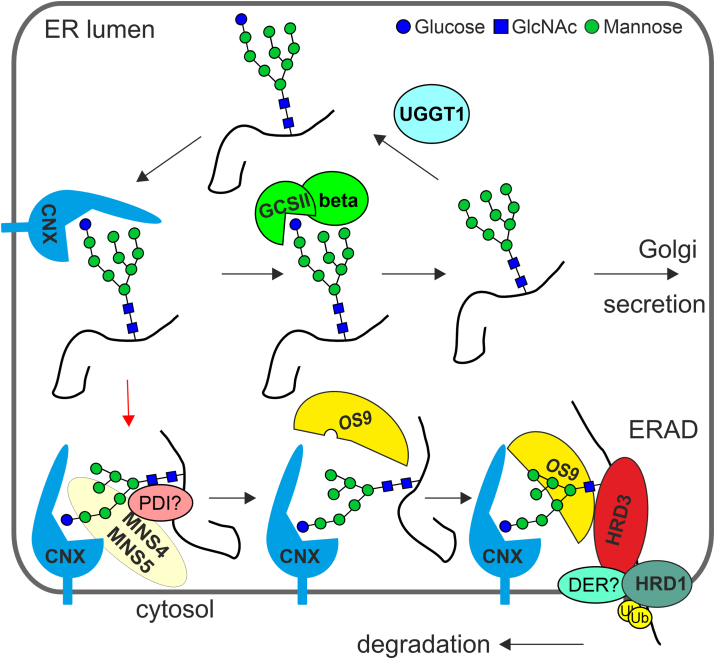
**Current model for the role of CNX and mannose trimming during ERAD of soluble misfolded glycoproteins.** Incompletely folded glycoproteins interact with CNX mainly *via* the monoglucosylated N-glycan. In the CNX cycle, glucosidase II (GCSII heterodimer consisting of alpha and beta subunits) removes the glucose residue, UGGT1 monitors the folding state and reglucosylates distinct folding intermediates or misfolded proteins which enables CNX to bind again to promote the folding with associated proteins (*e.g.* ERp57 in mammalian cells, not shown). The two α-mannosidases MNS4 and MNS5 together with interacting PDI-like proteins sense the misfolded ERAD substrate which diverts the protein away from GCSII and the CNX cycle. Trimming of the mannose residue on the C-branch generates the free α1,6-linked mannose that is bound by the carbohydrate binding protein OS9 that subsequently interacts with the canonical HRD1 ERAD complex for retrotranslocation, ubiquitination (Ub) and cytosolic degradation by the proteasome. CNX, calnexin; ERAD, ER-associated degradation; GCSII, α-glucosidase II; UGGT1, UDP-glucose:glycoprotein glucosyltransferase.

Proteins with a high degree of diversity are transported to the ER, where they are folded with the assistance of different factors, including enzymes and chaperones. During this process, the accumulation of folding intermediates and terminally misfolded proteins may occur. The precise mechanisms by which the ERQC and ERAD machineries distinguish between the various folding states and select terminally misfolded proteins for degradation remain unclear (12, 40, 41). Different models have been put forth to explain how futile folding attempts in the CNX/CRT cycle are terminated (42). One hypothesis is that severely misfolded glycoproteins are no longer reglucosylated by UGGT, thereby abolishing further interaction with CNX/CRT. This model is based on earlier reports suggesting that UGGT prefers nearly native-folded glycoproteins as substrates (43, 44, 45). More recent findings challenge this model and suggest that UGGT preferentially modifies N-glycans on extensively misfolded glycoproteins (12, 46, 47, 48). This reglucosylation activity implies that misfolded glycoproteins remain in the CNX/CRT cycle for a longer period before being released. Based on data from lectin (OS9 and CRT) binding and substrate specificities of enzymes like GCSII and UGGT it has been proposed that trimming of the α1,2-linked mannose from the C-branch locks glycoproteins in the CNX/CRT cycle (16, 49). This is consistent with our findings that mannose trimming does not have an effect on CNX binding (Fig. 10).

Previously, we reported small amounts of monoglucosylated N-glycans on misfolded BRI1-5 (19) which was confirmed independently by analysis of BRI1-5 N-glycans isolated from the *ebs5/hrd3/sel1l* single and *mns4 mns5* double mutant (8). In addition, the association of BRI1-5 with CNX has been demonstrated (27). Thus, both well-characterized glycoprotein ERAD substrates, SUBEX-C57Y and BRI1-5, interact specifically with CNX in a monoglucosylation-dependent manner. The underlying mechanism for binding of the ERAD substrates to CNX instead of CRT is currently unknown. CNX preferentially binds to membrane-associated glycoproteins, whereas CRT associates with soluble glycoproteins. The observed differences may be related to differences in subcellular localization of the lectins or preferred interaction of the ERAD membrane-proximal complex with the membrane-anchored CNX. Human EDEM1 has been demonstrated to specifically interact with CNX, and a misfolded α1-antitrypsin variant that is subjected to ERAD in mammalian cells has been shown to associate with CNX and not with CRT (14). These data are consistent with our findings and in favor of an important role of the CNX-EDEM/MNS4/MNS5 complex in targeting of misfolded glycoproteins to ERAD.

It is noteworthy that our findings reveal clear differences between the ERAD pathways in humans and plants. First, the overexpression of CNX has been observed to suppress ERAD in the presence of human EDEM (14) which is not the case for SUBEX-C57Y in plants. Second, human EDEM facilitates ERAD by releasing misfolded glycoproteins from CNX (14, 15). The present study shows that MNS4 promotes ERAD of SUBEX-C57Y, yet this occurs independently of release from CNX. The overexpression of either CNX or CRT did not impede ERAD in the presence of MNS4, and neither mannose trimming nor association with OS9 resulted in the disruption of SUBEX-C57Y binding to CNX. Third, abolished glycoprotein association with CNX accelerates ERAD in mammals (50), while it does not for SUBEX-C57Y in plants. An explanation for these differences could be that mammalian EDEMs do not only act on the C-branch of the N-glycan like MNS4/MNS5, but also on the A- and B-branches (17, 51, 52). In particular, the trimming of the mannose from the A-branch has been demonstrated to irreversibly remove the acceptor for UGGT1-mediated reglucosylation and CNX binding. In conclusion, the findings of this study highlight considerable mechanistic differences compared to mammals. Here, it is demonstrated that the free α1,6-mannose on the C-branch, and not the monoglucose on the A branch, is the dominant N-glycan signal that determines the fate of misfolded soluble glycoproteins. The inhibition of glucose trimming by CST, has been observed to result in the accelerated degradation of misfolded glycoproteins (53, 54). However, this effect has not been universally confirmed (14), and the findings for SUBEX-C57Y are most consistent with the effect of CST on influenza hemagglutinin, which resulted in aggregation and degradation (55).

Here, we observed an unexpected persistent interaction of CNX with the glycosylated ERAD substrate. A study in mammalian cells proposed the involvement of a dominant downstream ERAD sorting receptor to release ERAD substrates from CNX (12). However, no receptor with these properties has yet been identified in mammalian cells and the release mechanism is unknown. Notably, a persistent association of CNX with misfolded glycoproteins has been reported for ERAD-resistant mammalian proteins selected for ER-to-lysosome-associated degradation (56, 57). This alternative protein degradation pathway is utilized when the degradation of canonical ERAD substrates is prevented (58). It is possible that CNX may play a similar role in plants, but this has yet to be demonstrated.

In our model, at least one monoglucosylated N-glycan from an incompletely folded glycoprotein interacts with CNX which is in a complex with MNS4/MNS5. It is plausible that mannose trimming is gradual (mannose removal acting as a timer), and a misfolded glycoprotein with several N-glycans may undergo repeated cycles until the mannose residues are trimmed (Fig. 10). Upon processing by the α-mannosidase MNS4 or MNS5, the misfolded glycoprotein with CNX still bound to the monoglucosylated N-glycan is targeted to the HRD1 complex and subjected to retrotranslocation to the cytosol which is mediated with the help of CDC48. During the retrotranslocation the interaction with CNX is abrogated which could be caused by the CDC48-mediated complex disassembly and protein unfolding function (59). A block of glucose trimming (by CST or in the *knf-101* mutant) or premature removal of the glucose (by EndoM) both prevent CNX interaction which affects the solubility of the misfolded protein. This finding is consistent with the result of an earlier study with mammalian cells that demonstrated the role of UGGT1-dependent monoglucosylation of N-linked glycoproteins in promoting substrate solubility within the ER (34). The loss of CNX interaction may result in aggregation, consequently rendering the misfolded glycoproteins no longer a substrate for ERAD.

Although the knockout of both MNS4 and MNS5 is required to fully suppress the *Arabidopsis bri1-5* growth defect (19), recent data suggest that MNS4 and MNS5 have a client-specific function (20). This is corroborated by the evidence that MNS4 overexpression has a more pronounced effect on SUBEX-C57Y degradation and release from the CRT-mediated block of degradation than MNS5. The reason for the observed differences between MNS4 and MNS5 is currently unknown but may be related to the presence of different disordered regions at their C terminus (Fig. S9). MNS4 has a long-disordered domain at its C terminus, which is not present in MNS5. It is conceivable that this region is directly implicated in substrate binding or interaction with other ER-resident proteins that assist in client recognition and trigger the mannose trimming. EDEM3 has also been found to contain an intrinsically disordered domain at the C terminus and this region appears to modulate the efficiency and selectivity of substrate degradation (60). EDEM1 has intrinsically disordered domains located at the N terminus and C terminus. Deletion of the N-terminal domain abolishes EDEM1’s ability to bind and promote the degradation of ERAD substrates, even when the catalytic mannosidase domain remains intact (61). In mammals and yeast, EDEM and EDEM-like proteins form complexes with oxidoreductases such as PDI or ERdj5 (40, 62, 63, 64, 65, 66, 67, 68). The intrinsically disordered domains present in EDEM1 are required for ERdj5 binding (69). Similarly, it is conceivable that MNS4 and MNS5 have distinct interaction partners that regulate their substrate specificity. Further studies are required to elucidate the differences between *Arabidopsis* MNS4 and MNS5 and identify potential interaction partners that are crucial for their function in ERAD of misfolded glycoproteins in plants.

## Experimental procedures

### Plant material

*A. thaliana* was grown under long-day conditions (16 h light/8 h dark) at 22 °C with 50% relative humidity. T-DNA insertion line *os9*, the *mns4 mns5* double mutant and *knf-101* were described previously (19, 21, 38, 39). The *os9 cnx1 cnx2* triple mutant was generated by crossing of *os9* to SALK_083600 (*cnx1* knockout) and SALK_044381 (*cnx2* knockout) which have been described previously (27). Transgenic *Arabidopsis* expressing SUBEX-C57Y was available from a previous study (7) and GAUT4-GFP was generated by floral dipping as described previously (7). For inhibitor treatment, 7- to 9-day-old seedlings were incubated for 18 h in 0.5 × Murashige and Skoog liquid medium (Duchefa) supplemented with 1% (w/v) sucrose and 50 μM kifunensine (kif, Santa Cruz Biotechnology) and/or 100 μM CST, (Sigma-Aldrich). *N. benthamiana* were grown on soil under long-day conditions at 24 °C with 50% relative humidity. For inhibitor treatments in *N. benthamiana*, 20 μM CST, 20 μM kifunensine, or 20 μM bortezomib (BTZ, Sigma-Aldrich) were infiltrated 20 h prior to harvesting with 50 mM MES, 2 mM Na_3_PO_4_ 12H_2_O, into leaf areas that have previously been infiltrated with agrobacterial suspensions.

### Cloning of plasmid constructs

The SUBEX-C57Y-GFP expression vector p47-SUBEX-C57Y (mutated, glycosylated variant), the vector p47-SUBEX-WT for SUBEX-WT-GFP (nonmutated extracellular domain from *Arabidopsis* STRUBBELIG) expression, the vector p47-SUBEX-C57Y-NQ123 for SUBEX-C57Y-NQ123-GFP (mutated, nonglycosylated SUBEX-C57Y variant) expression, p46-NNN (GNTI-GFP, Golgi-marker) (70), p47-MNS4 (MNS4-GFP) and p47-MNS5 (MNS5-GFP) were described previously (19). Expression constructs p48-MUR3 (MUR3-RFP) (71), p48-MNS4 (MNS4-RFP), p48-MNS4-E376Q (MNS4-E376Q-RFP), p48-MNS5 (MNS5-RFP) (19), p31-OS9 (OS9-RFP), p20-OS9 (OS9-GFP) (21), p59-CRT2 (CRT2-RFP) (72), p110-CNX1 (RFP-CNX1) (72), and p45-CDC48-QQ (RFP-CDC48-QQ) (73) for RFP fusion protein expression and the BRI1 expression vector pPT8 (21) were all described previously. Expression vectors p74-SUBEX-C57Y (SUBEX-C57Y-GFP + YFP as expression control), p45-AtCDC48 (RFP-CDC48), p37-AtCDC48 (GFP-CDC48) and p37-AtCDC48-QQ (GFP-CDC48-QQ) were all described recently (24). The expression vector p79-CNX1 (GFP-CNX1) was generated by PCR amplification of *CNX1* (*AT5G61790*) from p110-CNX1 with primers CNX1_F/CNX1_R (Table S1). The *CNX2* (*AT5G07340*) gene was amplified from *Arabidopsis* Col-0 genomic DNA by PCR with CNX2_F/CNX2_R primers. The PCR product was XbaI and BamHI digested and cloned into p110 (72) to generate p110-CNX2 (RFP-CNX2). The *CRT1* (*AT1G56340*) coding region was amplified by PCR from *Arabidopsis* Col-0 cDNA with primers CRT1_F/CRT1_R, the PCR product was XbaI/BamHI digested and cloned into p59 (26) to generate p59-CRT1 (CRT1-RFP-HDEL). For MLO-1 (W162R amino acid exchange) (28) expression, the *Hordeum vulgare MLO* coding sequence with the indicated mutation was synthesized by Mr Gene (Mr Gene GmbH, Germany) (Table S2). The MLO-1 coding sequence was amplified by PCR with primers MLO1_F/MLO1_R, XbaI/BglII digested and cloned into p47 (74) to generate p47-MLO1 (MLO-1-GFP). The sequence encoding mouse EndoM was amplified by PCR from the GFP-EM plasmid (kindly provided by Christof Völker, University of Bonn, Germany) (75) using primers EndoM_F/EndoM_R. The PCR product was SpeI and BamHI digested and cloned into p48 to generate p48-EndoM (EndoM-RFP) or into p59 to generate p59-EndoM (EndoM-RFP-HDEL). For the p47-GAUT4 expression vector (GAUT4-GFP), the *Arabidopsis GAUT4* (*AT5G47780*) ORF was amplified by PCR with primers GAUT4_F/GAUT4_R. The PCR product was SpeI and BamHI digested and cloned into p47. The constructs for the SUBEX-C57Y retrotranslocation assay (SP-RFP-SUBEX-C57Y-NG11, NG10, and SP-NG10) were described previously (24). In vectors p46, p47, p48, p74, and p110 expression of the protein of interest is under the control of the *Arabidopsis ubiquitin 10* (*UBQ10*) promoter. In vectors pPT8, p31, p37, p45, p59, and p79 expression of the protein is under the control of the cauliflower mosaic virus 35S promoter.

### Confocal microscopy

For subcellular localization studies, liquid cultures of the agrobacterium strain UIA143 carrying one of the different expression constructs were diluted in infiltration buffer (50 mM MES, pH 5.6, 2 mM Na_3_PO_4_ 12H_2_O, 5 mg/ml D-glucose, and 0.1 mM acetosyringone [Sigma-Aldrich]) to an *A*_600_ of 0.1 to 0.2, unless stated otherwise. The suspension was infiltrated into leaf sections through stomata on the abaxial leaf side from 5-weeks-old *N. benthamiana* plants using a 1 ml syringe without a needle. High-resolution confocal images were acquired 2 days post infiltration on an upright Leica SP5 II confocal microscope using the Leica LAS AF software. GFP and RFP were excited with 488 and 561 nm laser lines, respectively, and signals were collected at 500 to 530 and 600 to 630 nm, respectively. Dual-color image acquisition of cells expressing both GFP and RFP was performed simultaneously (70).

### Cycloheximide treatment of *Arabidopsis* seedlings

*Arabidopsis* seedlings stably expressing SUBEX-C57Y-GFP under the control of the *A. ubiquitin 10* promoter were reported previously (7). Seeds were surface sterilized with 70% (v/v) ethanol containing 0.1% (v/v) Tween-20 and spread on 0.5× Murashige and Skoog medium (Duchefa) containing 0.8% (w/v) agar and 1% (w/v) sucrose. The Petri dishes were first incubated for 2 days in the dark at 4 °C, followed by incubation under long-day conditions at 22 °C in a growth chamber. Eight- to nine-day-old seedlings were transferred to six-well plates containing 0.5× Murashige and Skoog liquid medium with 100 μg/ml cycloheximide (CHX, Sigma-Aldrich) and incubated for the specified time period. The seedlings were removed, rinsed with water, briefly dried, and frozen in liquid nitrogen.

### Immunoblotting

Sections from infiltrated *N. benthamiana* leaves or transgenic *Arabidopsis* plants were harvested, frozen in liquid nitrogen, and ground in a mixer mill (Retsch) using stainless steel grinding balls. The homogenized plant material was resuspended (1 ml buffer per 500 mg of plant material) in protein extraction buffer containing 1 × PBS, pH 7.4, supplemented with 1% (v/v) Triton X-100 or 50 mM Tris–HCl, pH 8.0, 150 mM NaCl, 0.5 mM EDTA, 1% (v/v) IGEPAL CA-630 (Sigma-Aldrich), 0.5% (v/v) sodium deoxycholate (Sigma-Aldrich), 0.1% (v/v) SDS, and 1% (v/v) protease inhibitor cocktail (Sigma-Aldrich). The samples were cleared by centrifugation at 3500*g* for 5 min and 2 times centrifugation at 16,000*g* for 15 min. The clear supernatant was mixed with 3 × sample loading buffer, incubated at 95 °C for 5 min and subjected to SDS-PAGE followed by transfer of the proteins to an Amersham Protran Premium Western Blotting membrane (Cytiva). The membrane was blocked by incubation for 1 h in 5% (w/v) nonfat dry milk, and analysis was done using the following antibodies. Primary antibodies against GFP (1:2000 dilution, 11814460001, Roche), RFP (1:2000, 6g6, ChromoTek), BRI1 (1:5000, AS121859, Agrisera), CNX1/2 (1:2500, AS122365, Agrisera), alpha-tubulin (1:5000, T6074, Sigma-Aldrich) and actin (1:3000, A0480, Sigma-Aldrich) were commercially available. The antibody used to simultaneously detect endogenous CNX and CRT proteins in plants (1:10,000, AB00071, α-CRT/CNX) (76) was obtained from the plant antibody facility of the Ohio State University, OH. As secondary antibodies rabbit anti-mouse immunoglobulin G-horseradish peroxidase (IgG-HRP) conjugated antibody (1:10,000, A9044, Sigma-Aldrich), goat anti-rabbit IgG-HRP conjugated antibody (1:10,000, AS09602, Agrisera), or goat anti-rabbit IgG-HRP conjugated antibody (1:50,000, A0545, Sigma-Aldrich) were used. Membranes were developed with SuperSignal West Pico PLUS Chemiluminescent Substrate (Thermo Fisher Scientific), AgriseraECL Bright (Agrisera) or AgriseraECL SuperBright (Agrisera) and images from immunoblots were acquired using the Fusion XS (Vilber) or with X-ray films (Colenta). Subsequently, 0.1% (w/v) Ponceau S (Sigma-Aldrich) in 5% (v/v) acetic acid was used to stain the membranes. Densitometric analysis was performed using the Fusion Evolution-Capt software (Vilber) or from scanned images using Image J.

Data are represented by bar charts showing the mean ± SD of all data points. The numbers of replicates (n) are given in each figure legend. Statistical analysis (two tailed Student’s *t* test) was performed using GraphPad Prism version 10.0.1.

### Copurification of stably or transiently expressed ERAD components

Leaves of 5-week-old *N. benthamiana* plants were coinfiltrated with agrobacterial suspensions (*A*_600_ 0.1–0.2) carrying expression constructs for different proteins (*e.g.* SUBEX-C57Y, CRT1/CRT2, and CNX1/CNX2 variants). Two hundred milligrams infiltrated leaf material was harvested 2 days post infiltration, frozen in liquid nitrogen, and homogenized with a mixer mill. In addition, 500 μl of RIPA buffer (Sigma-Aldrich) supplemented with 0.5 mM EDTA and 1% (v/v) protease inhibitor cocktail was added, and the homogenized plant material was incubated on ice for 20 min; 500 μl 10 mM Tris–HCl, pH 7.5, 150 mM NaCl, and 0.5 mM EDTA were added followed by clarification at 3500*g* for 5 min at 4 °C and 2 to 4 times centrifugation at 16,000*g* for 15 min at 4 °C until a clear supernatant was obtained.

To enrich for GFP-tagged proteins, leaf extracts were incubated with 20 μl GFP-Trap (ChromoTek) agarose beads for 1 h at 4 °C with gentle rotation. After the incubation, the GFP-Trap beads were washed 3 times with ice-cold buffer (10 mM Tris–HCl, pH 7.5, 150 mM NaCl, and 0.5 mM EDTA). The agarose beads were collected by centrifugation at 1500*g* for 3 min at 4 °C and resuspended in 40 μl 2 × sample loading buffer. The suspension was transferred onto a Micro Bio-Spin chromatography column (Bio-Rad) and incubated for 2 min at 95 °C. Twenty microliters of 2 × sample loading buffer were added to the column, incubated for 2 min at 95 °C, and in total 60 μl eluate was collected by centrifugation at 1500*g* for 3 min and used for SDS-PAGE followed by immunoblot analysis.

### MS analysis of glycopeptides and glycans

For LC-ESI-MS/MS analysis of glycopeptides, SUBEX-C57Y-GFP was transiently expressed in *N. benthamiana* leaf epidermal cells and purified using GFP-Trap beads (ChromoTek) as described above. The purified proteins were separated by SDS-PAGE and stained with Coomassie Brilliant Blue. The bands corresponding to SUBEX-C57Y-GFP were excised from the gel, destained, S-alkylation of cysteine residues was performed using iodoacetamide, followed by enzymatic digestion with sequencing-grade trypsin (Promega) (77). For JBM (Sigma-Aldrich) digestion, trypsin-digested dried pellets were dissolved in ammonium formate (pH 4.4), 2 mM ZnCl_2_ and incubated with 0.8 U JBM (Sigma-Aldrich) for 16 h at 37 °C. The resulting peptides were separated using a nanoEase M/Z HSS T3 C18 column (100 Å, 1.8 μm, 300 μm × 150 mm, Waters) with 0.1% (v/v) formic acid in water as the aqueous mobile phase (solvent A). Peptides were eluted using a linear gradient from 1% to 40% solvent B (80% acetonitrile and 0.1% formic acid) over 40 min, followed by a 5 min gradient from 40% to 95% B to elute larger peptides, at a constant flow rate of 6 μl/min. Mass spectrometric detection was carried out on an Orbitrap Exploris 480 mass spectrometer (Thermo Fisher Scientific), equipped with a standard H-ESI source operated in positive ion mode using data-dependent acquisition. MS survey scans were acquired in the m/z range of 350 to 3200 Da, and the eight most intense precursor ions were selected for MS/MS fragmentation. Instrument calibration was performed using the Pierce FlexMix Calibration Solution (Thermo Fisher Scientific).

Putative glycopeptides were identified based on characteristic patterns of mass shifts corresponding to N-glycan compositions, including variable numbers of N-acetylhexosamine (HexNAc), hexose, deoxyhexose, and pentose residues attached to a common peptide backbone. Theoretical monoisotopic masses of the glycopeptides were calculated using custom spreadsheets based on the known masses of amino acids and monosaccharides. Manual data interpretation and glycopeptide identification were conducted using FreeStyle 1.8 (Thermo Fisher Scientific). Quantitative comparison of different glycoforms was performed based on the peak intensities of deconvoluted spectra. Glycoform annotation and data parsing were supported by the in-house developed software Glyco-parser (available at: https://github.com/lucaz88/Freestyle_parser).

For isomer analysis, N-glycans were released from glycopeptides by PNGase A (Proglycan) digestion and subsequently analyzed by porous graphitic carbon (PGC)-LC-ESI-MS on a Hypercarb column (0.32 × 150 mM, Thermo Fisher Scientific) coupled to an Ultimate 3000 (Dionex) capillary HPLC and a Q-TOF Ultima MS (Waters) as described in detail previously (78).

### Peptide mapping by LC-ESI-MS/MS

Agrobacterial suspensions carrying p47-SUBEX-C57Y and p47-SUBEX-WT, respectively, were coinfiltrated with Agrobacteria carrying p45-CDC48-QQ. For each purification, 600 mg of infiltrated leaf tissue was harvested 2 days after infiltration. The tissue was frozen in liquid nitrogen and then homogenized using a mixer mill. Two volumes of a buffer solution containing 10 mM Tris–HCl, pH 7.5, 150 mM NaCl, 0.5 mM EDTA, and 0.5% (v/v) IGEPAL CA-630, supplemented with 1% (v/v) protease inhibitor cocktail, were then added, after which the samples were incubated on ice for 20 min. The metal beads used for leaf homogenization were then removed by centrifugation at 3500*g* for 10 min at 4 °C, after which the samples were diluted with an equal volume of 10 mM Tris–HCl, pH 7.5, 150 mM NaCl, and 0.5 mM EDTA. The samples were cleared by centrifugation twice at 16,000*g* for 20 min at 4 °C and once at 40,000 *g* for 40 min at 4 °C. The GFP-Trap agarose beads were then washed and equilibrated with 10 mM Tris–HCl, pH 7.5, 150 mM NaCl, and 0.5 mM EDTA. Briefly, 22.5 μl of the beads were added to each sample, which was then incubated with gentle shaking for 60 min at 4 °C. After incubation, the beads were collected by centrifugation at 3500*g* for 4 min, then subjected to two washing steps using a buffer solution containing 10 mM Tris–HCl, pH 7.5, 150 mM NaCl, 0.5 mM EDTA, and 0.025% (v/v) IGEPAL CA-630, followed by one washing step using the same buffer solution. The beads were then transferred to Micro Bio-Spin chromatography columns (Bio-Rad) and washed twice with 600 μl of a buffer solution containing 10 mM Tris–HCl, pH 7.5, 150 mM NaCl, and 0.5 mM EDTA. For trypsin on-bead digestion, the agarose beads were incubated for 15 min at 60 °C with 10 mM dithiothreitol (Sigma-Aldrich) in a freshly prepared 100 mM NH_4_HCO_3_ buffer solution. For S-alkylation, 18 mM iodoacetamide (Sigma-Aldrich) in 100 mM NH_4_HCO_3_ buffer was added, after which the samples were incubated for 30 min in the dark. Then, 200 ng of sequencing grade modified trypsin (Promega) was added, and the samples were incubated with gentle shaking for 18 h at 37 °C. The peptides were isolated by centrifugation of the Micro Bio-Spin chromatography columns, then acidified by the addition of 20% (v/v) formic acid (final concentration 1%).

For MS analysis, the digested samples were separated and measured as described for the glycopeptides. The data files were analyzed using the PEAKS DB search tool (79) and searched against the indicated protein sequences derived from the *N. benthamiana* proteome NbeHZ1_proteome_1.0 (http://lifenglab.hzau.edu.cn/Nicomics/index.php).

## Data availability

Raw data files from MS analysis are available *via* ProteomeXchange with identifier PXD066844.

## Supporting information

This article contains supporting information.

## Conflict of interest

The authors declare that they have no conflicts of interest with the contents of this article.

## References

[1] Christianson J.C., Carvalho P. (2022). Order through destruction: how ER-associated protein degradation contributes to organelle homeostasis. EMBO J..

[2] Duan Z., Chen K., Yang T., You R., Chen B., Li J. (2023). Mechanisms of endoplasmic reticulum protein homeostasis in plants. Int. J. Mol. Sci..

[3] Zielinska D.F., Gnad F., Schropp K., Wiśniewski J.R., Mann M. (2012). Mapping N-glycosylation sites across seven evolutionarily distant species reveals a divergent substrate proteome despite a common core machinery. Mol. Cell.

[4] Strasser R. (2016). Plant protein glycosylation. Glycobiology.

[5] Aebi M., Bernasconi R., Clerc S., Molinari M. (2010). N-glycan structures: recognition and processing in the ER. Trends Biochem. Sci..

[6] Vaddepalli P., Fulton L., Batoux M., Yadav R.K., Schneitz K. (2011). Structure-function analysis of STRUBBELIG, an *Arabidopsis* atypical receptor-like kinase involved in tissue morphogenesis. PLoS One.

[7] Hüttner S., Veit C., Vavra U., Schoberer J., Dicker M., Maresch D. (2014). A context-independent N-glycan signal targets the misfolded extracellular domain of *arabidopsis* STRUBBELIG to endoplasmic-reticulum-associated degradation. Biochem. J..

[8] Zhang J., Xia Y., Wang D., Du Y., Chen Y., Zhang C. (2022). A predominant role of AtEDEM1 in catalyzing a rate-limiting demannosylation step of an *Arabidopsis* endoplasmic reticulum-associated degradation process. Front. Plant Sci..

[9] Williams D.B. (2006). Beyond lectins: the calnexin/calreticulin chaperone system of the endoplasmic reticulum. J. Cell Sci..

[10] Kozlov G., Gehring K. (2020). Calnexin cycle - structural features of the ER chaperone system. FEBS J..

[11] Strasser R. (2018). Protein quality control in the endoplasmic reticulum of plants. Annu. Rev. Plant Biol..

[12] Tannous A., Patel N., Tamura T., Hebert D.N. (2015). Reglucosylation by UDP-glucose:glycoprotein glucosyltransferase 1 delays glycoprotein secretion but not degradation. Mol. Biol. Cell.

[13] Helenius A., Trombetta E.S., Hebert D.N., Simons J.F. (1997). Calnexin, calreticulin and the folding of glycoproteins. Trends Cell Biol..

[14] Oda Y., Hosokawa N., Wada I., Nagata K. (2003). EDEM as an acceptor of terminally misfolded glycoproteins released from calnexin. Science.

[15] Molinari M., Calanca V., Galli C., Lucca P., Paganetti P. (2003). Role of EDEM in the release of misfolded glycoproteins from the calnexin cycle. Science.

[16] Stigliano I.D., Alculumbre S.G., Labriola C.A., Parodi A.J., D'Alessio C. (2011). Glucosidase II and N-glycan mannose content regulate the half-lives of monoglucosylated species *in vivo*. Mol. Biol. Cell.

[17] Ninagawa S., Okada T., Sumitomo Y., Kamiya Y., Kato K., Horimoto S. (2014). EDEM2 initiates mammalian glycoprotein ERAD by catalyzing the first mannose trimming step. J. Cell Biol..

[18] George G., Ninagawa S., Yagi H., Furukawa J.I., Hashii N., Ishii-Watabe A. (2021). Purified EDEM3 or EDEM1 alone produces determinant oligosaccharide structures from M8B in mammalian glycoprotein ERAD. Elife.

[19] Hüttner S., Veit C., Vavra U., Schoberer J., Liebminger E., Maresch D. (2014). *Arabidopsis* class I α-mannosidases MNS4 and MNS5 are involved in endoplasmic reticulum-associated degradation of misfolded glycoproteins. Plant Cell.

[20] Sun X., Guo C., Ali K., Zheng Q., Wei Q., Zhu Y. (2022). A non-redundant function of MNS5: a class I alpha-1,2 mannosidase, in the regulation of endoplasmic reticulum-associated degradation of misfolded glycoproteins. Front. Plant Sci..

[21] Hüttner S., Veit C., Schoberer J., Grass J., Strasser R. (2012). Unraveling the function of *Arabidopsis thaliana* OS9 in the endoplasmic reticulum-associated degradation of glycoproteins. Plant Mol. Biol..

[22] Hong Z., Kajiura H., Su W., Jin H., Kimura A., Fujiyama K. (2012). Evolutionarily conserved glycan signal to degrade aberrant brassinosteroid receptors in *Arabidopsis*. Proc. Natl. Acad. Sci. U. S. A..

[23] Su W., Liu Y., Xia Y., Hong Z., Li J. (2012). The *Arabidopsis* homolog of the mammalian OS-9 protein plays a key role in the endoplasmic reticulum-associated degradation of misfolded receptor-like kinases. Mol. Plant.

[24] Schoberer J., Vavra U., Shin Y.J., Grunwald-Gruber C., Strasser R. (2025). Elucidation of the late steps in the glycan-dependent ERAD of soluble misfolded glycoproteins. Plant J..

[25] Su W., Liu Y., Xia Y., Hong Z., Li J. (2011). Conserved endoplasmic reticulum-associated degradation system to eliminate mutated receptor-like kinases in Arabidopsis. Proc. Natl. Acad. Sci. U. S. A..

[26] Schoberer J., König J., Veit C., Vavra U., Liebminger E., Botchway S.W. (2019). A signal motif retains arabidopsis ER-α-mannosidase I in the *cis*-Golgi and prevents enhanced glycoprotein ERAD. Nat. Commun..

[27] Hong Z., Jin H., Tzfira T., Li J. (2008). Multiple mechanism-mediated retention of a defective brassinosteroid receptor in the endoplasmic reticulum of *Arabidopsis*. Plant Cell.

[28] Müller J., Piffanelli P., Devoto A., Miklis M., Elliott C., Ortmann B. (2005). Conserved ERAD-like quality control of a plant polytopic membrane protein. Plant Cell.

[29] Liu L., Cui F., Li Q., Yin B., Zhang H., Lin B. (2011). The endoplasmic reticulum-associated degradation is necessary for plant salt tolerance. Cell Res..

[30] Zhang J., Wu J., Liu L., Li J. (2020). The crucial role of demannosylating asparagine-linked glycans in eradicating misfolded glycoproteins in the endoplasmic reticulum. Front. Plant Sci..

[31] Jin H., Yan Z., Nam K., Li J. (2007). Allele-specific suppression of a defective brassinosteroid receptor reveals a physiological role of UGGT in er quality control. Mol. Cell.

[32] Ji Z., Li H., Peterle D., Paulo J.A., Ficarro S.B., Wales T.E. (2022). Translocation of polyubiquitinated protein substrates by the hexameric CDC48 ATPase. Mol. Cell.

[33] Molinari M., Galli C., Vanoni O., Arnold S.M., Kaufman R.J. (2005). Persistent glycoprotein misfolding activates the glucosidase II/UGT1-driven calnexin cycle to delay aggregation and loss of folding competence. Mol. Cell.

[34] Ferris S.P., Jaber N.S., Molinari M., Arvan P., Kaufman R.J. (2013). UDP-glucose:glycoprotein glucosyltransferase (UGGT1) promotes substrate solubility in the endoplasmic reticulum. Mol. Biol. Cell.

[35] Hardt B., Volker C., Mundt S., Salska-Navarro M., Hauptmann M., Bause E. (2005). Human endo-alpha1,2-mannosidase is a Golgi-resident type II membrane protein. Biochimie.

[36] Fujimoto K., Kornfeld R. (1991). Alpha-glucosidase II-deficient cells use endo alpha-mannosidase as a bypass route for N-linked oligosaccharide processing. J. Biol. Chem..

[37] Roth J., Ziak M., Zuber C. (2003). The role of glucosidase II and endomannosidase in glucose trimming of asparagine-linked oligosaccharides. Biochimie.

[38] Furumizu C., Komeda Y. (2008). A novel mutation in *knopf* uncovers the role of alpha-glucosidase I during post-embryonic development in *Arabidopsis thaliana*. FEBS Lett..

[39] Farid A., Pabst M., Schoberer J., Altmann F., Glössl J., Strasser R. (2011). *Arabidopsis thaliana* alpha1,2-glucosyltransferase (ALG10) is required for efficient N-glycosylation and leaf growth. Plant J..

[40] Liu Y.C., Fujimori D.G., Weissman J.S. (2016). HTM1p-PDI1p is a folding-sensitive mannosidase that marks N-glycoproteins for ER-associated protein degradation. Proc. Natl. Acad. Sci. U. S. A..

[41] Adams B.M., Oster M.E., Hebert D.N. (2019). Protein quality control in the endoplasmic reticulum. Protein J..

[42] Molinari M. (2007). N-glycan structure dictates extension of protein folding or onset of disposal. Nat. Chem. Biol..

[43] Caramelo J.J., Castro O.A., de Prat-Gay G., Parodi A.J. (2004). The endoplasmic reticulum glucosyltransferase recognizes nearly native glycoprotein folding intermediates. J. Biol. Chem..

[44] Ritter C., Quirin K., Kowarik M., Helenius A. (2005). Minor folding defects trigger local modification of glycoproteins by the ER folding sensor GT. EMBO J..

[45] D'Alessio C., Caramelo J.J., Parodi A.J. (2010). UDP-Glc:Glycoprotein glucosyltransferase-glucosidase II, the ying-yang of the ER quality control. Semin. Cell Dev. Biol..

[46] Calles-Garcia D., Yang M., Soya N., Melero R., Menade M., Ito Y. (2017). Single-particle electron microscopy structure of UDP-glucose:Glycoprotein glucosyltransferase suggests a selectivity mechanism for misfolded proteins. J. Biol. Chem..

[47] Roversi P., Marti L., Caputo A.T., Alonzi D.S., Hill J.C., Dent K.C. (2017). Interdomain conformational flexibility underpins the activity of UGGT, the eukaryotic glycoprotein secretion checkpoint. Proc. Natl. Acad. Sci. U. S. A..

[48] Adams B.M., Canniff N.P., Guay K.P., Larsen I.S.B., Hebert D.N. (2020). Quantitative glycoproteomics reveals cellular substrate selectivity of the ER protein quality control sensors UGGT1 and UGGT2. Elife.

[49] Kuribara T., Usui R., Totani K. (2021). Glycan structure-based perspectives on the entry and release of glycoproteins in the calnexin/calreticulin cycle. Carbohydr. Res..

[50] Moore S.E., Spiro R.G. (1993). Inhibition of glucose trimming by castanospermine results in rapid degradation of unassembled major histocompatibility complex class I molecules. J. Biol. Chem..

[51] Olivari S., Cali T., Salo K., Paganetti P., Ruddock L., Molinari M. (2006). EDEM1 regulates ER-associated degradation by accelerating de-mannosylation of folding-defective polypeptides and by inhibiting their covalent aggregation. Biochem. Biophys. Res. Commun..

[52] Avezov E., Frenkel Z., Ehrlich M., Herscovics A., Lederkremer G. (2008). Endoplasmic reticulum (ER) mannosidase I is compartmentalized and required for N-glycan trimming to Man5-6GlcNAc2 in glycoprotein ER-associated degradation. Mol. Biol. Cell.

[53] Kearse K.P., Williams D.B., Singer A. (1994). Persistence of glucose residues on core oligosaccharides prevents association of TCR alpha and tcr beta proteins with calnexin and results specifically in accelerated degradation of nascent TCR alpha proteins within the endoplasmic reticulum. EMBO J..

[54] Ayalon-Soffer M., Shenkman M., Lederkremer G.Z. (1999). Differential role of mannose and glucose trimming in the ER degradation of asialoglycoprotein receptor subunits. J. Cell Sci..

[55] Hebert D.N., Foellmer B., Helenius A. (1996). Calnexin and calreticulin promote folding, delay oligomerization and suppress degradation of influenza hemagglutinin in microsomes. EMBO J..

[56] Fregno I., Fasana E., Solda T., Galli C., Molinari M. (2021). N-glycan processing selects ERAD-resistant misfolded proteins for ER-to-lysosome-associated degradation. EMBO J..

[57] Forrester A., De Leonibus C., Grumati P., Fasana E., Piemontese M., Staiano L. (2019). A selective ER-phagy exerts procollagen quality control via a calnexin-FAM134B complex. EMBO J..

[58] Fasana E., Fregno I., Galli C., Solda T., Molinari M. (2024). ER-to-lysosome-associated degradation acts as failsafe mechanism upon ERAD dysfunction. EMBO Rep..

[59] Stolz A., Hilt W., Buchberger A., Wolf D.H. (2011). CDC48: a power machine in protein degradation. Trends Biochem. Sci..

[60] Manica G., Ghenea S., Munteanu C.V.A., Martin E.C., Butnaru C., Surleac M. (2021). EDEM3 domains cooperate to perform its overall cell functioning. Int. J. Mol. Sci..

[61] Marin M.B., Ghenea S., Spiridon L.N., Chiritoiu G.N., Petrescu A.J., Petrescu S.M. (2012). Tyrosinase degradation is prevented when EDEM1 lacks the intrinsically disordered region. PLoS One.

[62] Gauss R., Kanehara K., Carvalho P., Ng D.T., Aebi M. (2011). A complex of PDI1p and the mannosidase HTM1p initiates clearance of unfolded glycoproteins from the endoplasmic reticulum. Mol. Cell.

[63] Pfeiffer A., Stephanowitz H., Krause E., Volkwein C., Hirsch C., Jarosch E. (2016). A complex of HTM1 and the oxidoreductase PDI1 accelerates degradation of misfolded glycoproteins. J. Biol. Chem..

[64] Shenkman M., Ron E., Yehuda R., Benyair R., Khalaila I., Lederkremer G.Z. (2018). Mannosidase activity of EDEM1 and EDEM2 depends on an unfolded state of their glycoprotein substrates. Commun. Biol..

[65] Yu S., Ito S., Wada I., Hosokawa N. (2018). ER-resident protein 46 (ERP46) triggers the mannose-trimming activity of er degradation-enhancing alpha-mannosidase-like protein 3 (EDEM3). J. Biol. Chem..

[66] George G., Ninagawa S., Yagi H., Saito T., Ishikawa T., Sakuma T. (2020). EDEM2 stably disulfide-bonded to TXNDC11 catalyzes the first mannose trimming step in mammalian glycoprotein erad. Elife.

[67] Zhao D., Wu X., Rapoport T.A. (2025). Initiation of ERAD by the bifunctional complex of MNL1/HTM1 mannosidase and protein disulfide isomerase. Nat. Struct. Mol. Biol..

[68] Hitchman C.J., Lia A., Chiritoiu G.N., Munteanu C.V.A., Ortigosa J.R., Ghenea S. (2025). Structure and function of the EDEM:PDI ERAD checkpoint complex. bioRxiv.

[69] Lamriben L., Oster M.E., Tamura T., Tian W., Yang Z., Clausen H. (2018). EDEM1's mannosidase-like domain binds erad client proteins in a redox-sensitive manner and possesses catalytic activity. J. Biol. Chem..

[70] Schoberer J., Liebminger E., Vavra U., Veit C., Castilho A., Dicker M. (2014). .The transmembrane domain of N-acetylglucosaminyltransferase I is the key determinant for its Golgi subcompartmentation. Plant.

[71] Schoberer J., Liebminger E., Vavra U., Veit C., Grünwald-Gruber C., Altmann F. (2019). The Golgi localization of GNTI requires a polar amino acid residue within its transmembrane domain. Plant Physiol..

[72] Göritzer K., Goet I., Duric S., Maresch D., Altmann F., Obinger C. (2020). Efficient N-glyco*s*ylation of the heavy chain tailpiece promotes the formation of plant-produced dimeric IgA. Front. Chem..

[73] Shin Y.J., König-Beihammer J., Vavra U., Schwestka J., Kienzl N.F., Klausberger M. (2021). N-glycosylation of the SARS-CoV-2 receptor binding domain is important for functional expression in plants. Front. Plant Sci..

[74] Strasser R., Stadlmann J., Svoboda B., Altmann F., Glössl J., Mach L. (2005). Molecular basis of N-acetylglucosaminyltransferase I deficiency in *Arabidopsis thaliana* plants lacking complex N-glycans. Biochem. J..

[75] Gallo G.L., Valko A., Aramburu S.I., Etchegaray E., Völker C., Parodi A.J. (2018). Abrogation of glucosidase I-mediated glycoprotein deglucosylation results in a sick phenotype in fission yeasts: model for the human MOGS-CDG disorder. J. Biol. Chem..

[76] Jin H., Hong Z., Su W., Li J. (2009). A plant-specific calreticulin is a key retention factor for a defective brassinosteroid receptor in the endoplasmic reticulum. Proc. Natl. Acad. Sci. U. S. A..

[77] Stadlmann J., Pabst M., Kolarich D., Kunert R., Altmann F. (2008). Analysis of immunoglobulin glycosylation by LC-ESI-MS of glycopeptides and oligosaccharides. Proteomics.

[78] Pabst M., Grass J., Toegel S., Liebminger E., Strasser R., Altmann F. (2012). Isomeric analysis of oligomannosidic N-glycans and their dolichol-linked precursors. Glycobiology.

[79] Zhang J., Xin L., Shan B., Chen W., Xie M., Yuen D. (2012). PEAKS DB: de novo sequencing assisted database search for sensitive and accurate peptide identification. Mol. Cell Proteomics.

