# Supplementary material for: Mannose trimming is the dominant signal for the release of misfolded glycoproteins from ER quality control
Source: J Biol Chem. 2025 Aug 28;301(10):110649. doi: 10.1016/j.jbc.2025.110649 (PMC12494556; doi:10.1016/j.jbc.2025.110649)
Supplement: Supporting Figures and Tables [file mmc1.docx]

**Supporting Information**

**Mannose trimming is the dominant signal for the release of misfolded glycoproteins from ER quality control**

Yun-Ji Shin^1^, Ulrike Vavra^1^, Daniel Maresch^2^, Clemens Grünwald-Gruber^2^, Richard Strasser^1*^

^1^Institute of Plant Biotechnology and Cell Biology, Department of Biotechnology and Food Sciences, BOKU University; Vienna, Austria

^2^Core Facility Mass Spectrometry, BOKU University; Vienna, Austria

*To whom correspondence may be addressed: [richard.strasser@boku.ac.at](mailto:richard.strasser@boku.ac.at)

**Running title**

Mannose trimming in ERAD

**Key words**

Cell biology, endoplasmic reticulum (ER), endoplasmic‐reticulum‐associated protein degradation (ERAD), glycoprotein, N-linked glycosylation, plant, protein degradation

**Supplementary** **Tables**

**Table S1. Primer sequences used in this study.**

| Primer Name | Sequence (5’-3’) |
| --- | --- |
| CNX1_F | TATATCTAGAGACGATCAAACGGTTCTGTATG |
| CNX1_R | TATAGGATCCCTAATTATCACGTCTCGGTTGCC |
| CNX2_F | TATATCTAGAGACGACCAAACGATCCTGTATGAA |
| CNX2_R | TATAGGATCCCTAACTCTCACGCCTCGTCTGCC |
| CRT1_F | TATATCTAGAATGGCGAAACTAAACCCTAAATTC |
| CRT1_R | TATAAGATCTGAGCTCGTCATGGGCGGCATCGGT |
| EndoM_F | TATAACTAGTATGGCAAAATTTCGAAGAAGGA |
| EndoM_R | TATAGGATCCTGAAGCAGGCTGCTGTTGATCC |
| GAUT4_F | TATAACTAGTATGATGGTGAAGCTTCGCAATCTTG |
| GAUT4_R | TATAGGATCCAGGATTGATGTTGCATTCTCTGA |
| MLO1_F | TATATCTAGAATGTCGGACAAAAAAGGG |
| MLO1_R | TATAAGATCTTAGAGAAGCGTAATCTGGAACATC |

**Table S2. Synthetic DNA sequence used in this study.**

| Name | DNA Sequence |
| --- | --- |
| MLO-1 | TCTAGAATGTCGGACAAAAAAGGGGTGCCGGCGCGGGAGCTGCCGGAGACGCCGTCGTGGGCGGTGGCGG TGGTCTTCGCCGCCATGGTGCTCGTGTCCGTCCTCATGGAACACGGCCTCCACAAGCTCGGCCATTGGTTC CAGCACCGGCACAAGAAGGCCCTGTGGGAGGCGCTGGAGAAGATGAAGGCGGAGCTCATGCTGGTGGGCT TCATATCCCTGCTCCTCATCGTCACGCAGGACCCCATCATCGCCAAGATATGCATCTCCGAGGATGCCGC CGACGTCATGTGGCCCTGCAAGCGCGGCACCGAGGGCCGCAAGCCCAGCAAGTACGTTGACTACTGCCCG GAGGGCAAGGTGGCGCTCATGTCCACGGGCAGCTTGCACCAGCTGCACGTCTTCATCTTCGTGCTCGCGG TCTTCCATGTCACCTACAGCGTCATCACCATAGCTCTAAGCCGTCTCAAAATGAGAACATGGAAGAAAAG GGAGACAGAGACCACCTCCTTGGAATACCAGTTCGCAAATGATCCTGCACGGTTCCGGTTCACGCACCAG ACGTCGTTCGTGAAGCGCCACCTGGGCCTCTCCAGCACCCCTGGCATCAGATGGGTGGTGGCCTTCTTCA GGCAGTTCTTCAGGTCAGTCACCAAGGTGGACTACCTGACCTTGAGGGCAGGCTTCATCAACGCGCATTT GTCGCAAAACAGCAAGTTCGACTTCCACAAGTACATCAAGAGGTCGATGGAGGACGACTTCAAGGTCGTC GTCGGCATCAGCCTCCCGCTGTGGGGTGTGGCGATCCTCACCCTCTTCCTTGACATCAATGGGGTTGGCA CGCTCATCTGGATTTCTTTCATCCCTCTCGTGATCCTCTTGTGTGTTGGAACCAAGCTGGAGATGATCAT CATGGAGATGGCCCTGGAGATCCAGGACCGGGCGAGCGTCATCAAGGGGGCCCCCGTGGTCGAGCCCAGC AACAAGTTCTTCTGGTTCCACCGCCCCGACTGGGTCCTCTTCTTCATACACCTGACGTTGTTCCAGAACG CGTTTCAGATGGCGCATTTTGTGTGGACAGTGGCCACGCCCGGCTTGAAGAAATGCTACCACACGCAGAT CGGGCTGAGCATCATGAAGGTGGTGGTGGGGCTAGCTCTCCAGTTCCTCTGCAGCTATATGACCTTCCCC CTCTACGCGCTCGTCACACAGATGGGATCAAACATGAAGAGGTCCATCTTCGACGAGCAGACGTCCAAGG CGCTCACCAACTGGCGGAACACGGCCAAGGAGAAGAAGAAAGTCCGAGACACGGACATGCTGATGGCTCA GATGATCGGCGACGCAACACCGAGCCGAGGCTCGTCGCCGATGCCGAGCCGGGGCTCATCACCCGTGCAC CTGCTTCACAAGGGCATGGGGCGGTCGGACGACCCCCAGAGCGCGCCCACCTCGCCAAGGACCCAGCAGG AGGCTAGGGACATGTACCCGGTTGTGGTGGCGCACCCGGTGCACAGACTAAATCCTAACGACAGGAGGAG GTCCGCCTCGTCGTCGGCCCTCGAAGCCGACATCCCCAGTGCAGATTTTTCCTTCAGCCAGGGAGGATCC TACCCATACGACGTTCCTGACTATGCGTCACTCTACCCCTATGACGTACCGGATTATGCATCCCTATATC CGTATGATGTTCCAGATTACGCTTCTCTATGAAGATCT |

**Table S3. Peptide intensities of GFP-Trap co-purified native CNX and CRT proteins from *N. benthamiana.***

| Protein | #1 | #2 | #3 | Mean | SD |
| --- | --- | --- | --- | --- | --- |
| SUBEX-C57Y | 3,26E+09 | 6,41E+09 | 4,54E+09 | 4,74E+09 | 1,58E+09 |
| CNX | 4,26E+08 | 7,68E+08 | 5,32E+08 | 5,75E+08 | 1,75E+08 |
| CRT | 5,25E+06 | 3,10E+06 | 2,87E+06 | 3,74E+06 | 1,31E+06 |
|  |  |  |  |  |  |
| Protein | #1 | #2 | #3 | MW | SD |
| SUBEX-WT | 4,45E+09 | 3,00E+09 | 1,35E+09 | 2,93E+09 | 1,55E+09 |
| CNX | 8,39E+06 | 4,43E+06 | 7,66E+06 | 6,83E+06 | 2,11E+06 |
| CRT | 3,18E+05 | 4,02E+05 | 7,48E+04 | 2,65E+05 | 1,70E+05 |

Peptide signal intensities from three (#1 to #3) biological replicates are shown. SUBEX-C57Y-GFP was GFP-Trap purified from infiltrated *N. benthamiana* leaves and digested with trypsin. The signal intensities of co-purified endogenous *N. benthamiana* CNX and CRT peptides are shown (upper panel). SUBEX-WT-GFP expression, GFP-Trap purification followed by trypsin digestion was used as a control (lower panel). See Excel file for further details of the MS analysis.

**Supplementary Figures**

**
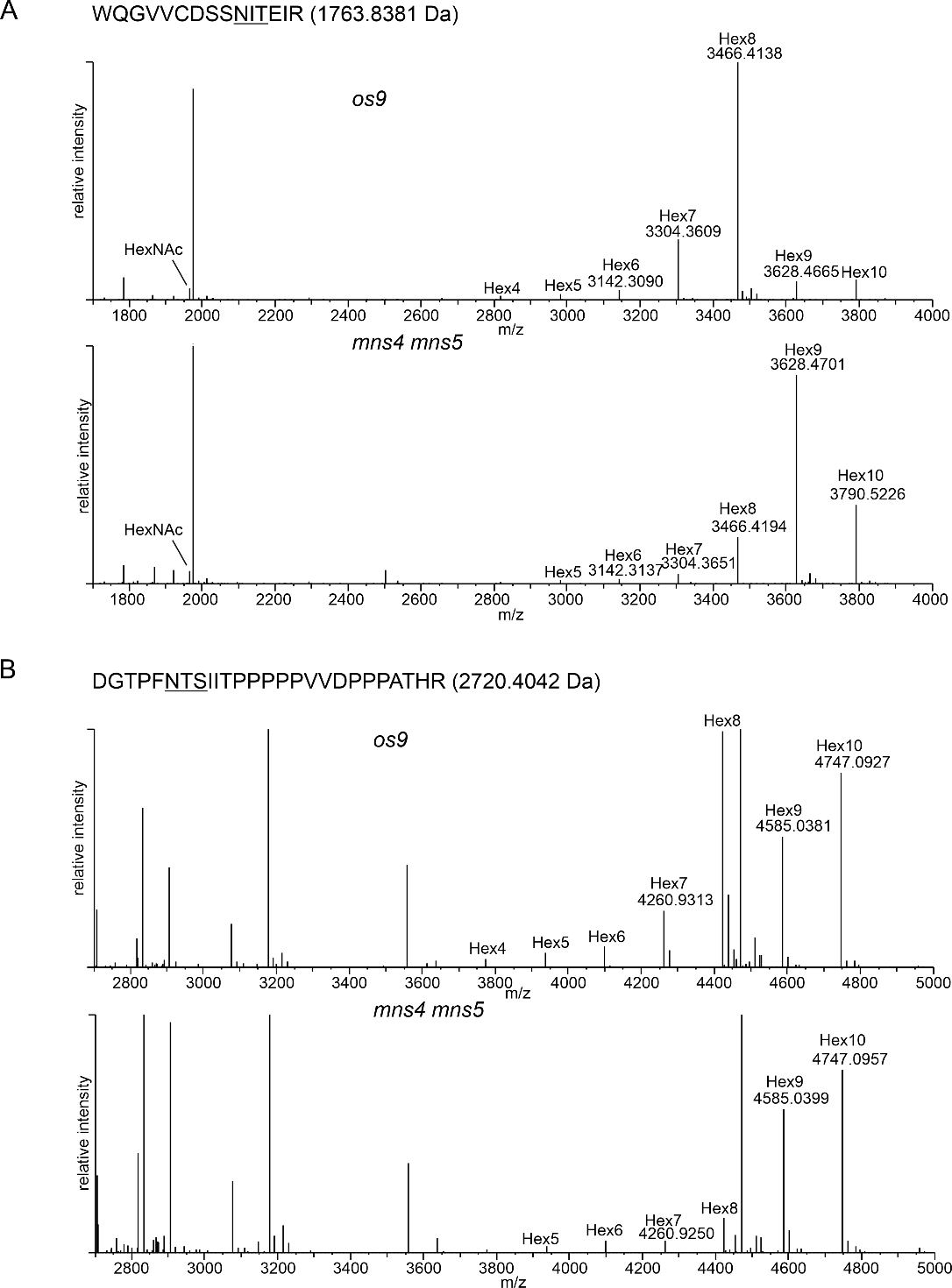
**

**Figure S1.** **LC-ESI-MS analysis of glycopeptides 1 and 3 from SUBEX-C57Y expressed in *A. thaliana* *os9* and *mns4 mns5* mutant plants.** Purified SUBEX-C57Y-GFP was subjected to proteolytic digestion and the indicated glycopeptides were analysed by LC-ESI-MS. (A) SUBEX-C57Y glycopeptide 1. (B) SUBEX-C57Y glycopeptide 3. The N-glycosylation site is underlined. The most abundant peaks (> 1 %) corresponding to N-glycan structures are labelled. Hex4 to Hex10 refers to Hexose4HexNAc2 to Hexose10HexNAc2 N-glycans present on the glycopeptides.

**
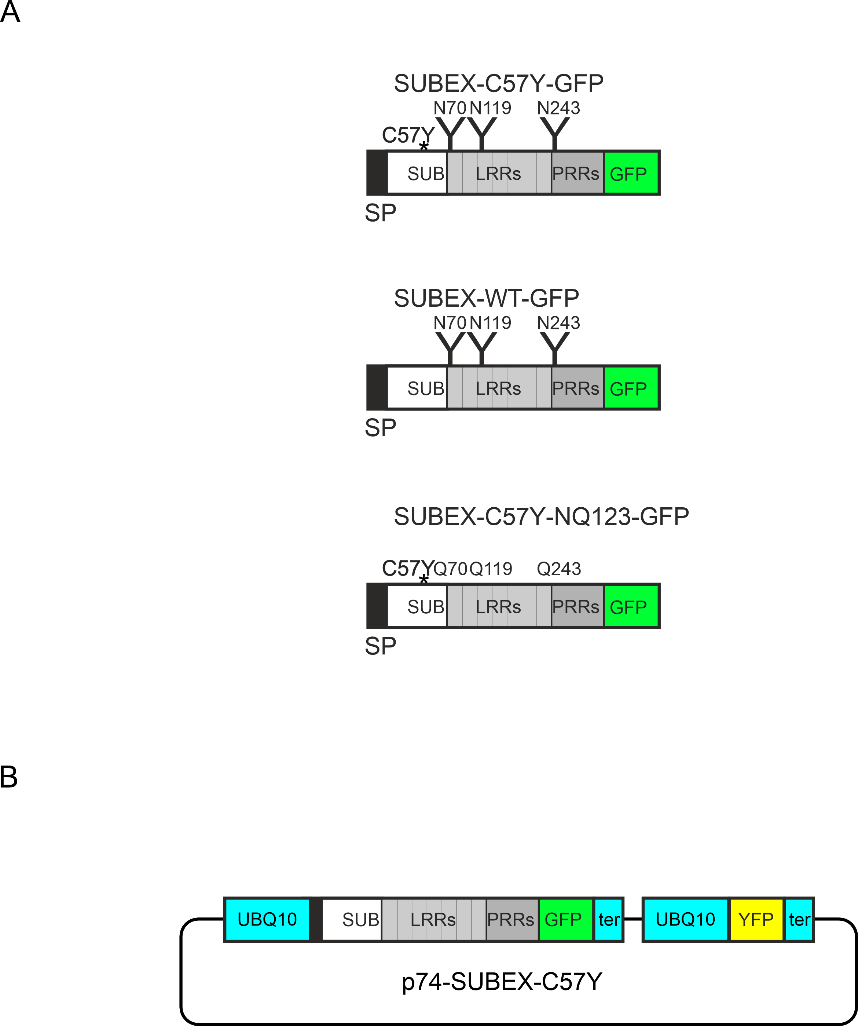
**

**Figure S2. Schematic illustration of the SUBEX protein variants and the SUBEX-C57Y expression vector carrying the YFP expression cassette.** (A) Illustration of different SUBEX variants. The C57Y mutation in the extracellular domain of STRUBBELIG (SUBEX) is indicated by an asterisk. N-glycosylation sites are represented by ‘Y’ and their amino acid positions are depicted. SP, STRUBBELIG signal peptide, SUB, STRUBBELIG-domain; LRRs, leucine-rich repeats; PRRs, proline-rich repeats. SUBEX-WT-GFP is glycosylated and non-mutated, in SUBEX-C57Y-NQ123-GFP all three N-glycosylation sites are mutated by replacing asparagine with glutamine. (B) p74-SUBEX-C57Y expression vector carrying the SUBEX-C57Y-GFP and YFP expression cassettes under the *Arabidopsis* *ubiquitin 10* (*UBQ10*) promoter. SUBEX-C57Y carries the endogenous signal peptide. YFP lacks a signal peptide and is present in the cytosol.

**
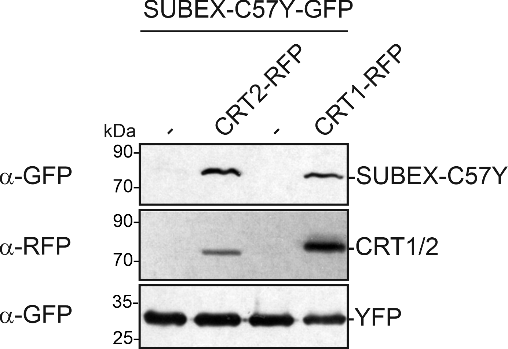
**

**Figure S3. Effect of CRT1 and CRT2 expression on SUBEX-C57Y levels.** Both CRT1-RFP and CRT2-RFP expression increase the amount of SUBEX-C57Y-GFP. The indicated proteins were transiently expressed in *N. benthamiana* and total protein extracts were subjected to immunoblotting. YFP detection is used as a control.

**
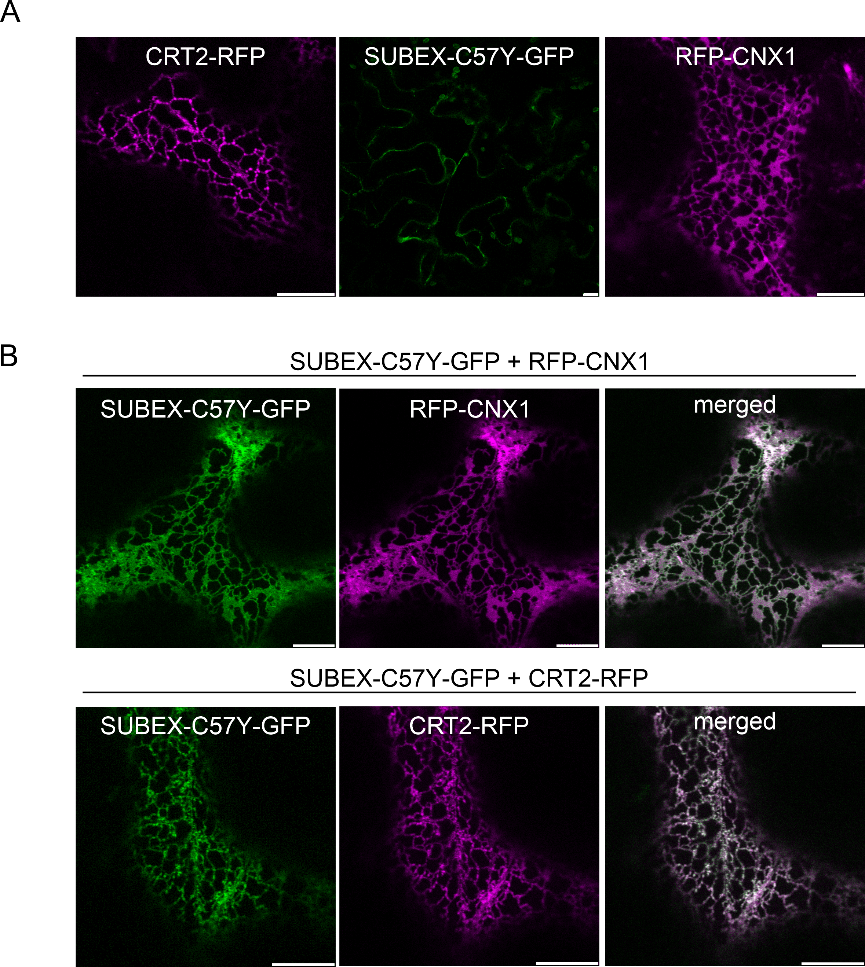
**

**Figure S4. Subcellular localization of CRT2-RFP, RFP-CNX1 and SUBEX-C57Y-GFP.** (A) CRT2-RFP (OD_600_ = 0.02), SUBEX-C57Y-GFP (OD_600_ = 0.20) and RFP-CNX1 (OD_600_ = 0.05) were transiently expressed in *N. benthamiana* leaves. 2 days post infiltration, leaf epidermal cells were analysed using confocal microscopy. (B) Co-localisation of SUBEX-C57Y-GFP with RFP-CNX1 and SUBEX-C57Y-GFP with CRT2-RFP. Scale bar = 10 µm.

**
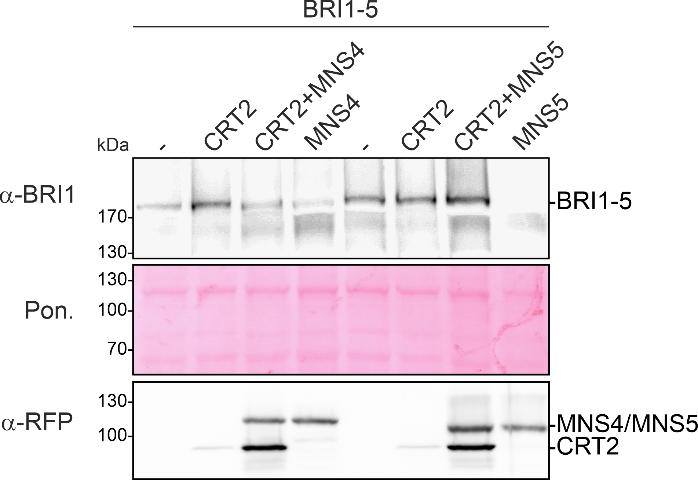
**

**Figure S5. Immunoblot analysis showing MNS4 and MNS5 activity with BRI1-5.** BRI1-5 was transiently expressed in *N. benthamiana* without any additional protein (-), with CRT2-RFP (CRT2), with CRT2-RFP and MNS4-RFP (CRT2+MNS4) or with MNS4-RFP (MNS4) alone. BRI1-5 was expressed without any additional protein (-), with CRT2-RFP (CRT2), with CRT2-RFP and MNS5-RFP (CRT2+MNS5) or with MNS5-RFP (MNS5) alone.

**
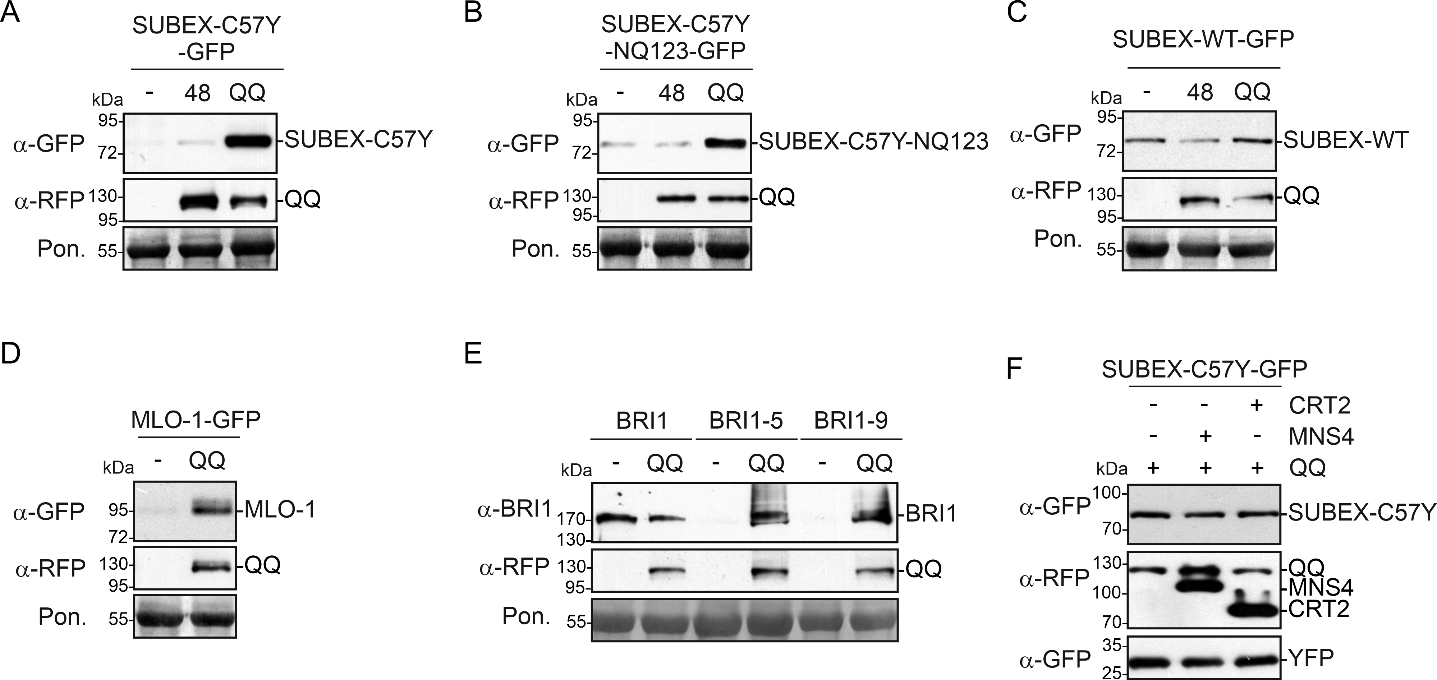
**

**Figure S6. The catalytically inactive AAA ATPase CDC48-QQ blocks the degradation of misfolded proteins.** (A-E) SUBEX-C57Y-GFP, the non-glycosylated variant SUBEX-C57Y-NQ123-GFP, the non-mutated variant SUBEX-WT-GFP, MLO-1-GFP, BRI1 wild-type (BRI1) and BRI1 mutant variants (BRI1-5 and BRI1-9) were transiently expressed in *N. benthamiana* leaves without (-) any additional protein, with RFP-CDC48 (48) or with RFP-CDC48-QQ (QQ) and analyzed by immunoblotting. Ponceau S (Pon.) staining is used as a loading control. (F) SUBEX-C57Y-GFP was expressed with RFP-CDC48-QQ (QQ), RFP-CDC48-QQ and MNS4-RFP (QQ+MNS4) or RFP-CDC48-QQ and CRT2-RFP (QQ+CRT2). YFP detection is used as a control.

**
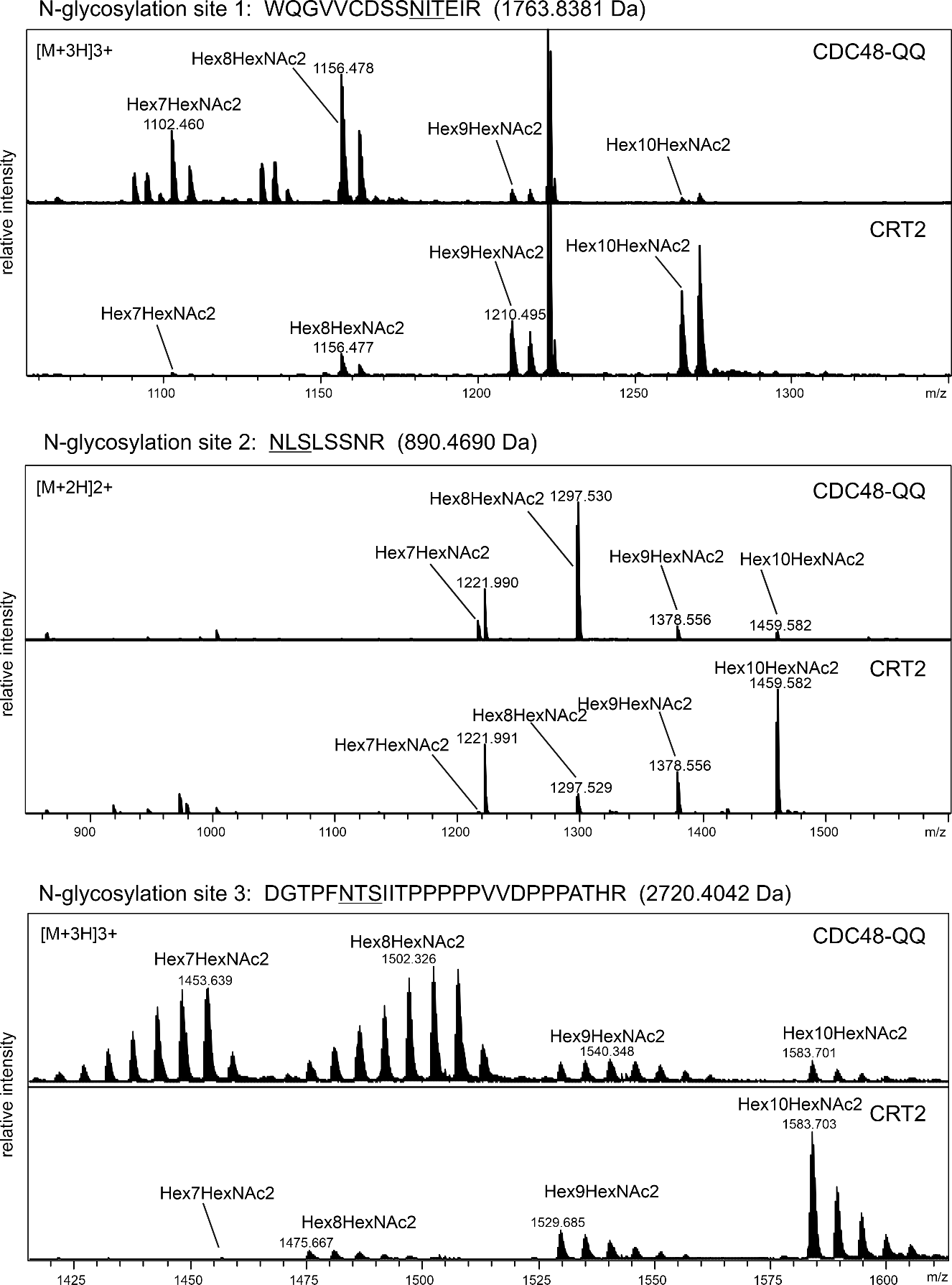
**

**Figure S7. MS spectra of the three SUBEX-C57Y glycopeptides.** CRT2 or CDC48-QQ was co-expressed to block the degradation of SUBEX-C57Y-GFP. SUBEX-C57Y-GFP was purified, subjected to trypsin digestion and glycopeptides were analyzed by LC-ESI-MS. Peaks corresponding to [M+2H]^2+^ or [M+3H]^3+^ ions are shown.

**
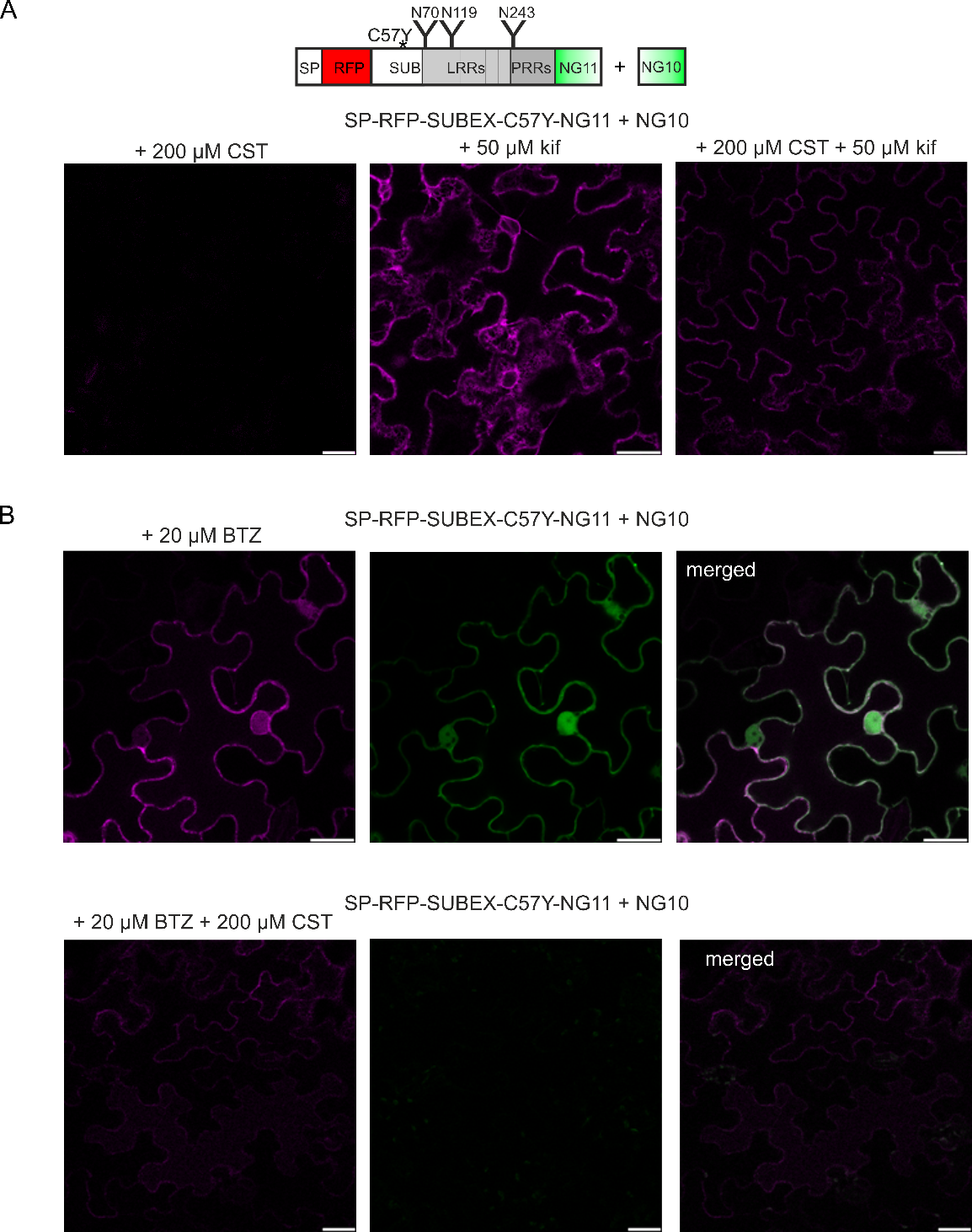
**

**Figure S8. Castanospermine (CST) reduces the ER levels of the ERAD substrate and interferes with ERAD.** (A) Illustration of the co-expressed proteins for the retrotranslocation assay using split mNeonGreen (NG11+NG1-10). SP-RFP-SUBEX-C57Y-NG11 was co-expressed with NG10 in *N. benthamiana* leaf epidermal cells in the presence of CST, kif or CST and kif. While kif causes an increase of the RFP signal in the ER indicating a block of ERAD, this effect is suppressed by CST. (B) Retrotranslocation of SP-RFP-SUBEX-C57Y-NG11 is observed by the green mNeonGreen signal in the cytosol and nucleus. BTZ was infiltrated to block the proteasome which is required to block the degradation of the retrotranslocated protein. Retrotranslocation of SP-RFP-SUBEX-C57Y-NG11 is blocked in the presence of CST (no specific mNeonGreen signal detected). Scale bars = 10 μm.


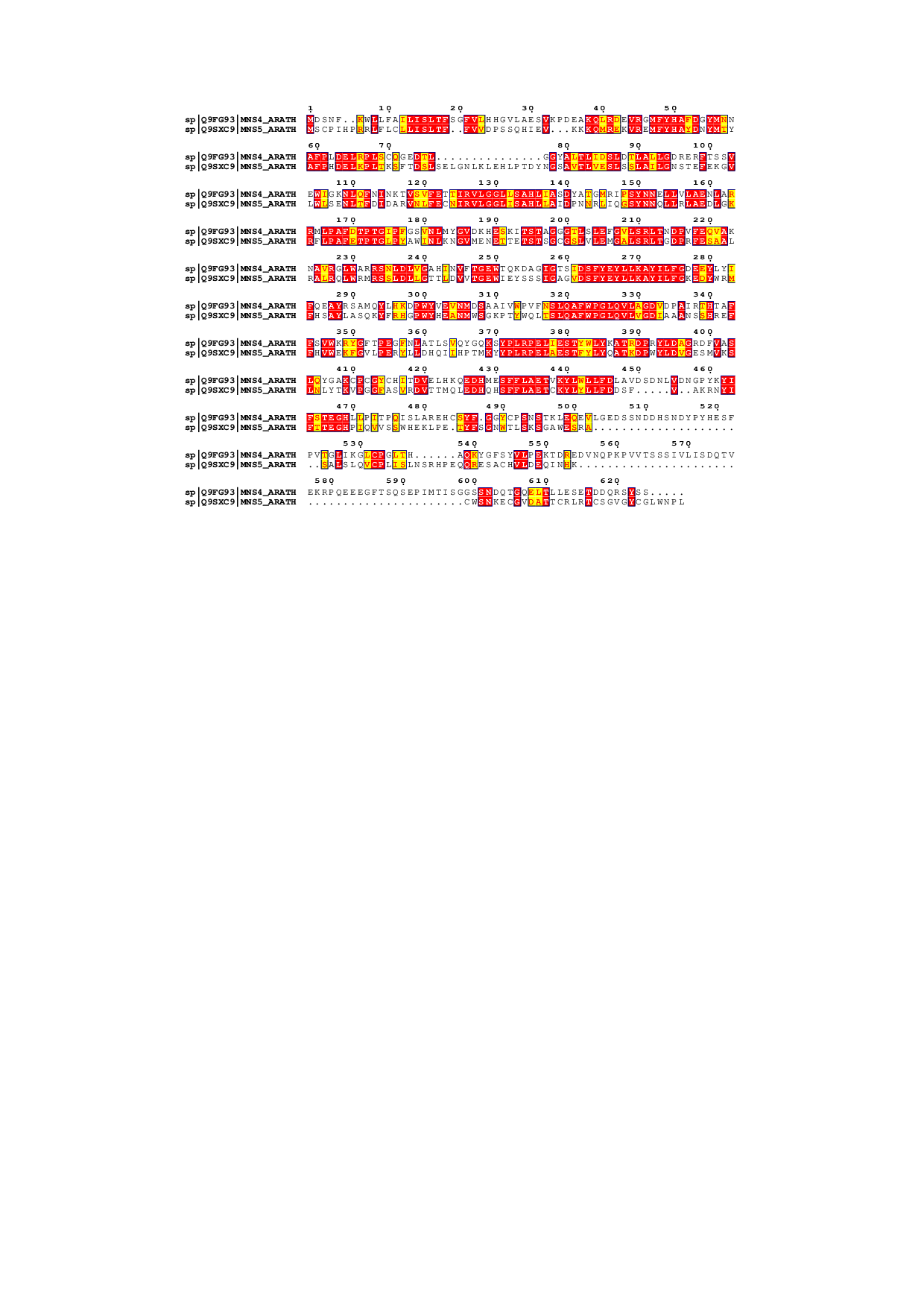
A

B MNS4 MNS5


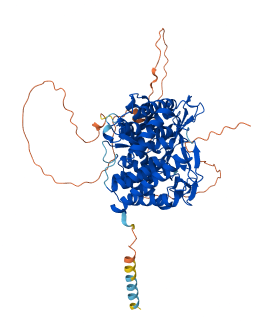

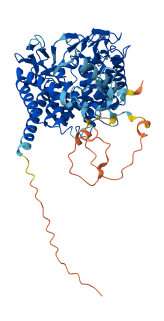


**Figure S9. *Arabidopsis* MNS4 and MNS5 amino acid sequence alignment and predicted structures.** (A) The alignment was done using Expresso (<https://tcoffee.crg.eu/apps/tcoffee/index.html>). The result file was then imported into ESPript 3.0 (<https://espript.ibcp.fr/ESPript/cgi-bin/ESPript.cgi>) to display the aligned sequences. Identical amino acid residues are shown in red, similar residues are shown in yellow. (B) Alphafold models of MNS4 (AF-Q9FG93-F1-v4, <https://alphafold.ebi.ac.uk/entry/Q9FG93>) and MNS5 (AF-Q9SXC9-F1-v4, <https://alphafold.ebi.ac.uk/entry/Q9SXC9>).
